# Optimizing Resistance Training for Sprint and Endurance Athletes: Balancing Positive and Negative Adaptations

**DOI:** 10.1007/s40279-024-02110-4

**Published:** 2024-10-07

**Authors:** Bas Van Hooren, Per Aagaard, Anthony J. Blazevich

**Affiliations:** 1 Department of Nutrition and Movement Sciences, NUTRIM Institute of Nutrition and Translational Research in Metabolism, Maastricht University Medical Centre+, Universiteitssingel 50, Maastricht, NL 6229 ER The Netherlands; 2 Department of Sports Science and Clinical Biomechanics, University of Southern Denmark, Odense, Denmark; 3 Centre for Human Performance, School of Medical and Health Sciences, Edith Cowan University, Joondalup, Australia

## Abstract

Resistance training (RT) triggers diverse morphological and physiological adaptations that are broadly considered beneficial for performance enhancement as well as injury risk reduction. Some athletes and coaches therefore engage in, or prescribe, substantial amounts of RT under the assumption that continued increments in maximal strength capacity and/or muscle mass will lead to improved sports performance. In contrast, others employ minimal or no RT under the assumption that RT may impair endurance or sprint performances. However, the morphological and physiological adaptations by which RT might impair physical performance, the likelihood of these being evoked, and the training program specifications that might promote such impairments, remain largely undefined. Here, we discuss how selected adaptations to RT may enhance or impair speed and endurance performances while also addressing the RT program variables under which these adaptations are likely to occur. Specifically, we argue that while some myofibrillar (muscle) hypertrophy can be beneficial for increasing maximum strength, substantial hypertrophy can lead to macro- and microscopic adaptations such as increases in body (or limb) mass and internal moment arms that might, under some conditions, impair both sprint and endurance performances. Further, we discuss how changes in muscle architecture, fiber typology, microscopic muscle structure, and intra- and intermuscular coordination with RT may maximize speed at the expense of endurance, or maximize strength at the expense of speed. The beneficial effect of RT for sprint and endurance sports can be further improved by considering the adaptive trade-offs and practical implications discussed in this review.

**Graphical abstract:**

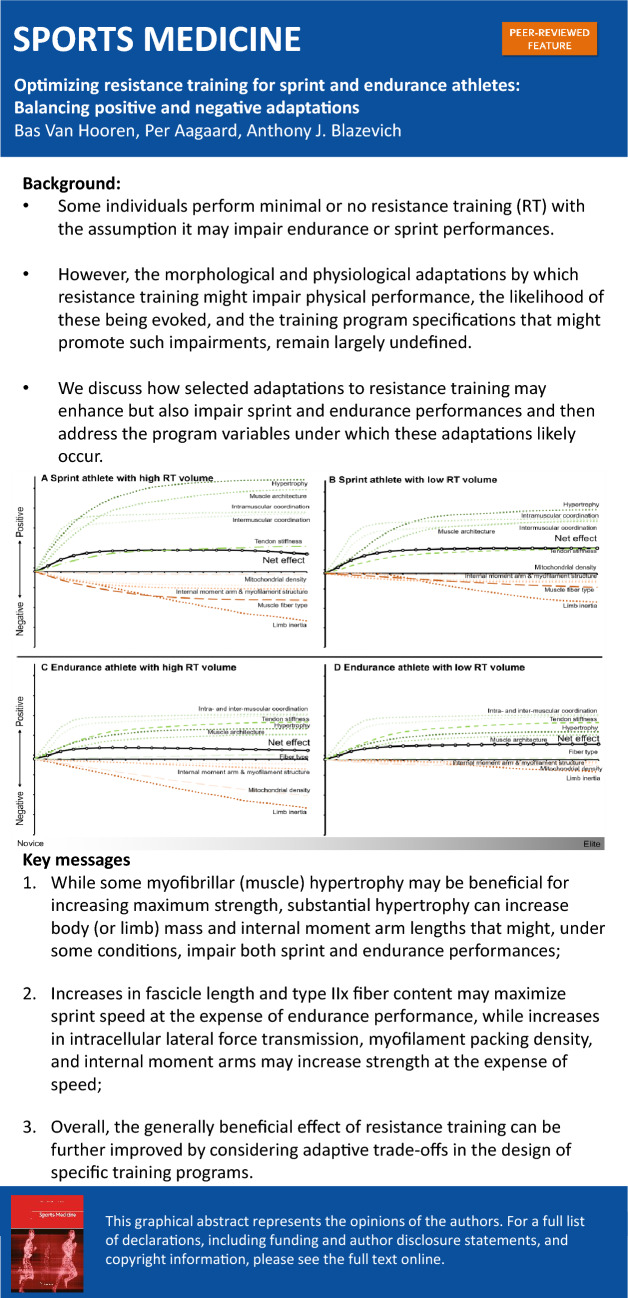

## Key Points

**Table:** 

While some myofibrillar (muscle) hypertrophy can be beneficial for increasing maximum strength, substantial hypertrophy can lead to macro- and microscopic adaptations such as increases in body (or limb) mass and internal moment arms that might, under some conditions, impair both sprint and endurance performance.
Training-induced increases in fascicle length and type IIx fiber content may maximize sprint speed at the expense of endurance performance, while increases in intracellular lateral force transmission, myofilament packing density, and internal moment arms may increase strength at the expense of maximum movement speed.
Overall, these findings suggest that the generally beneficial effect of resistance training can be further improved by considering these adaptive trade-offs in the design of specific training programs.

## Introduction

Resistance training (RT) is commonly used as a supplement to sport-specific training with the goal of enhancing performance and/or reducing injury risk. Indeed, RT promotes various neuromuscular adaptations that may influence these outcomes, including decreased motor unit recruitment thresholds, increased maximum motor unit firing rates, altered (optimized) intermuscular coordination, as well as changes in muscle architecture, fiber type, tendon stiffness, and extracellular matrix construct, with each adaptation varying according to specifications of the training program including load [1–3], volume [4, 5], and possibly contraction mode [6, 7].

As acknowledged in a recent position statement [8], muscle hypertrophy is also often a desirable outcome of RT in athletic populations because increased muscle mass is often associated with increased maximum force production, at least in trained individuals [9–13]. In lesser trained individuals, the relationship between increases in skeletal muscle mass and maximum force or sports performance is often less apparent—mainly (but not exclusively) due to a varying contribution of neural and other factors to increases in maximum force production—although generally positive associations are observed [14]. Moreover, increases in muscle mass have been associated with improved sports performance outcomes in some [15–17], but not all, studies [18–20]. Muscle hypertrophy may also offer the benefit of increased whole-body mass or inertia, which may be useful in some contact sports (especially during player collision) including combat and martial art sports. Based on the often-positive associations between muscle mass and strength or performance, some practitioners (athletes, coaches) impose substantive volumes of RT, often with a direct focus on skeletal muscle mass gain, to optimize performance in line with the adage that one “*can't go wrong getting strong*” or “*bigger is better.*”

In contrast, others believe that RT may not always induce beneficial adaptations [21–25] and therefore recommend using only little or no RT [21, 22, 24, 26–28]. For example, approximately half of the US Olympic marathon qualifiers, and 15% of international level cyclists reportedly use no RT at all [24, 27], and there are numerous other reports of (elite) athletes using minimal-to-no RT [29–31]. Reasons for the use of little or no RT include the assumption that RT decreases physical performance, or because it increases injury risk [25]. However, the resistance training-induced morphological and physiological adaptations that might impair performance or increase injury risk, the likelihood of these adaptations to be evoked, and the training program specifications that may promote such impairments, remain largely undefined. As a result, some—particularly endurance-oriented—athletes may incorrectly refrain from using RT in their regular training schedule because of its presumed negative impact on endurance performance or injury risk [23–25]. Moreover, individuals who incorporate RT may do so in ways that are suboptimal for performance enhancement or injury risk reduction [32–34] (i.e., low-load, high repetition exercise regimes [21, 23, 26]). By contrast, other athletes—particularly in sprint-type or explosive-type sport disciplines—may overuse RT under the assumption that continued increments in maximal strength and/or muscle mass will translate into more marked increases in sprint speed, etc. However, this assumption has been challenged by studies showing that higher levels of maximal strength and/or muscle mass are not always positively associated with greater sprint speeds [18–20] and in some instances may be negatively associated with sprint speed or other measures of explosive performance [35–37].

The present review aims to provide a synopsis of important adaptations to RT and then considers their potential beneficial and detrimental impacts on endurance and speed performances, using middle- and long-distance and sprint running as the primary examples. We also discuss the likelihood of selected beneficial and detrimental adaptations occurring to a substantial level (at least within our current understanding) and highlight some trade-offs between adaptations favoring endurance versus speed or strength, or favoring speed versus strength adaptations. Finally, we provide guidelines for minimizing detrimental and maximizing beneficial adaptations for specific movement qualities. This information will be of use to athletes, researchers, clinicians, and coaches to optimize RT exercise prescriptions for sports that require rapid force production such as sprint running, repeated submaximal force applications with minimum energy cost such as distance running, or with combinations of both, such as in many field, court, and ball sports.

## Neuromuscular Adaptations

### Macroscopic Structural and Architectural Adaptations

#### Muscle Hypertrophy

An increase in muscle size (i.e., muscle hypertrophy) is arguably the best known adaptation to RT and it is thought to reflect hypertrophy of individual muscle fibers, which in turn contributes to the increase in whole muscle size [38–40]. In contrast, the contribution of an increased muscle fiber number (i.e., hyperplasia) to muscle hypertrophy is unlikely given their post-mitotic state, with recent findings suggesting that the higher muscle fiber number observed in RT individuals in some cross-sectional studies (e.g., [41]) may reflect the regeneration of damaged (branched) fibers instead of the creation of de novo muscle fibers [42]. Fiber hypertrophy can in turn result from increased sarcoplasmic (e.g., extracellular fluid accumulation) or myofibrillar content. A larger myofibrillar content can reflect an increase in the size of existing myofibrils or an increase in the number of myofibrils, with recent findings indicating an important role for both mechanisms in muscle fiber hypertrophy, although an increase in the number of myofibrils was more important than the increase in size [43]. Similarly a comparison of long-term RT and untrained individuals also showed a greater number of myofibrils in long-term RT individuals [41]. Regardless, both mechanisms (i.e., increase in myofibril size or number) will directly contribute to an increased maximum force production [44, 45]. An increase in myofibrillar content of selected muscles may therefore be beneficial for sprint performance by increasing force producing capability, which in turn can lead to a greater production of propulsive impulse and work [46–49]. Potential benefits of an increased myofibrillar content may also be observed for endurance performance due to a lower relative force requirement and hence lower energy cost related to recruiting and activating myofibrils. Specifically, while enlarged muscle fibers will increase energy cost of the hypertrophied fiber itself by increasing the total number of active cross-bridges, the number of action potentials required by the whole muscle will be lower as fewer fibers need to be activated to produce a given force (due to fiber hypertrophy), thus reducing the energy cost of ion channel activation [50, 51]. Nevertheless, there are several biomechanical (e.g., inertia) and structural reasons (e.g., changes in fiber type) why (excessive) hypertrophy could also be detrimental to both sprint and endurance performance, as discussed in the following subsections.

##### Muscle Mass: A Trade-Off Between Strength and Endurance

Endurance athletes may disengage with RT under the assumption that muscle hypertrophy might meaningfully increase both limb and total body mass, and this could in turn increase the energy cost of movement. Indeed, body mass is the primary determinant of the energetic cost of running, with a larger body mass being strongly associated with a higher oxygen or energy cost [52–55]. As an example of the total effect of body and limb mass, a 1 kg increase in body mass is associated with a ~ 1–1.5% increase in oxygen cost in running (based on re-analyses of data from [56, 57] and [52]). Part of the increased energy cost stems from the extra work required to accelerate (raise) the body upward against gravity in each running step (i.e., external work), to which is added the muscle work done to swing the limbs (i.e., internal work), and the work done to overcome the muscle’s greater internal inertia during the phases of muscle shortening [58]. A final (small) increase in energy cost arises from the increase in aerodynamic resistance due to increases in body surface area with increases in body mass (see Appendix B in [59]).

Mechanistically, a larger body mass requires more energy to be produced to sustain a given running velocity (increased kinetic energy: 1/2 m × *v*^2^, where *m* is mass and *v* is the velocity) or to carry it a given distance (increased work: force × distance) because the larger mass requires a higher force production from muscles during the stance phase of running. Further, more energy is required to move the body upwards against gravity at each step in running because the gravitational potential energy gained is a linear function of mass, gravitational acceleration, and the height (vertical displacement of the body center of mass) gained in each running step. Alternatively, if mass is decreased, then an individual will bounce higher for the same energy input or bounce the same height with less energy input. Because the total change in mechanical energy (defined as kinetic energy + potential energy) dictates how we move, and as both factors are dependent on body mass, more muscle work (force production) is needed as mass increases. Increased contractile force production in turn requires higher motor unit discharge rates and/or increased motor unit recruitment to activate additional muscle fibers (see e.g. [7]), which further increase energy cost [50, 55].

In addition to its effect on whole body mass, muscle hypertrophy can also increase local and remote limb moments of inertia [i.e., which makes it harder to spin a segment about its own axis as well as an external (remote) axis such as the lower limb (shank) relative to the hip joint, respectively]. This angular-inertial effect will increase the energy cost of leg or arm angular acceleration during both flight and stance phases of running. Indeed, it follows from Newton’s second law (force = mass × acceleration, and thus joint torque = limb moment of inertia × limb angular acceleration) that a larger segment mass, and/or increased moment of inertia, requires a higher force or torque production for a given magnitude of segment acceleration (i.e., to reach a given limb velocity at specific phases of the running stride). Additionally, increased limb mass will be particularly detrimental to movement economy when it is added more distally in the limbs as opposed to proximally due to the increase in radius of gyration, which exponentially impacts limb moment of inertia (*I* = *mk*^2^, where *I* is the moment of inertia, *m* is the mass, and *k* is the radius of gyration). For example, adding mass distally to the legs (i.e. using heavy-type running shoes) increases energy cost considerably more than an equal mass added to the trunk (i.e., 4.5% for every 500 g on each leg but 1% by adding mass to the trunk; [60]). As a result, a larger mass (or limb circumference as a proxy of mass) of distal limb segments has been associated with a decreased running economy and poorer running performance in high-level runners [61, 62]. An additional consideration is that while a larger proximal muscle mass can increase whole body power output and thus contribute positively to production of the work needed to sustain a given running speed, a larger distal muscle mass will be less beneficial for enhancing the overall power output because distal muscles (e.g., calf muscles) typically perform less work by muscle contraction, but rather rely more on elastic mechanisms (tendon recoil) to produce power (i.e., increasing the rate at which concentric work is produced (e.g., [63–65]), see also Sect. 2.4). This results in a steep trade-off between increasing power output through muscle mass gain and increases in total limb inertia. In summary, while additional muscle mass might be beneficial to enhance propulsive capacity in runners (in particular in the case of proximal muscle hypertrophy), both an increased body mass per se and the addition of distal limb mass are likely to increase the energy cost of movement to negatively affect movement economy, in turn potentially impairing endurance performance.

Since running economy is an important factor influencing distance running performance [66], a low total body mass—as often observed in elite endurance runners—should be beneficial. Similarly, team sports athletes cover a substantial distance during a match (e.g., 8–12 km for soccer players [67]; ~ 8 km for elite Gaelic Football players [68]; ~ 13 km for elite Australian Rules football players [69, 70]), so running economy, including during accelerations, decelerations and changes of direction, may also be an important component of performance in certain team sports [71, 72]. While a larger body mass may be beneficial to increase short-distance acceleration capacity [16], and during contact or collisions with opponents due to the possession of greater inertia and momentum [73], it can also be detrimental to the ability to cover a large in-game running distance and the ability to perform repeated sprints with short recovery periods [74]. This trade-off needs to be considered on an individual basis and in relation to each athlete’s playing position, tactical contributions, and preferences.

The combination of resistance and endurance training is known as concurrent training. The likelihood that substantial additional body mass will be gained by endurance athletes and team sports athletes when RT is added to the normal training program is low because only minor or no increases in body mass have been reported after RT combined with concurrent endurance training [75–79] or team sports practice [71]. Specifically, in recreational to elite endurance athletes, the mean increase in body mass among multiple concurrent training studies was ~ 1 kg relative to an endurance-only group following 12–18 weeks of training with 2–3 RT sessions and a similar or greater number of endurance training sessions performed per week [75, 77, 79–81]. The absence of changes in body mass in concurrent training studies may partially result from these studies often reporting a decrease in fat mass in conjunction with an increase in lean body mass (e.g., larger vastus lateralis muscle fiber cross-sectional area [82]), resulting in a lack of net body mass change and thus an increase in the strength-to-body mass ratio [75, 77, 81]. Moreover, endurance training activates pathways (AMPK) that are known to inhibit muscle hypertrophy signaling pathways (mTOR) [83–86], thus also attenuating increases in fiber size with endurance training, in particular for running [87]. Therefore, while muscle hypertrophy following RT could theoretically impair endurance performance as a result of increases in body mass and increased myocellular diffusion distances (discussed below), the magnitude of these effects is likely to be small or negligible in a typical concurrent training setting in which RT is performed two to three times per week with a small-to-moderate training volume per session (e.g. two to three lower-body exercises each performed in two to three sets with four to six repetitions per set and a loading intensity of 85% one-repetition maximum [1-RM]), in line with previous suggestions [88]. Nevertheless, larger volumes of RT may increase muscle mass also in a concurrent setting, which potentially may cause the strength of the athlete to increase at the expense of endurance performance. Indeed, it seems that previously untrained individuals tend to show greater relative gains in muscle fiber cross-sectional area in response to concurrent training protocols than more extensively trained individuals [89], and it has been suggested that this increase in fiber cross-sectional area may attenuate the increase in maximum oxygen uptake in a concurrent training setting [89]. Similarly, a large RT volume may also negatively impact training quality during a subsequent endurance session, thereby potentially attenuating endurance adaptations [90].

##### Muscle Mass and Moment Arms: Trade-Offs Between Strength–Speed and Speed–Endurance

**Fig. 1:**
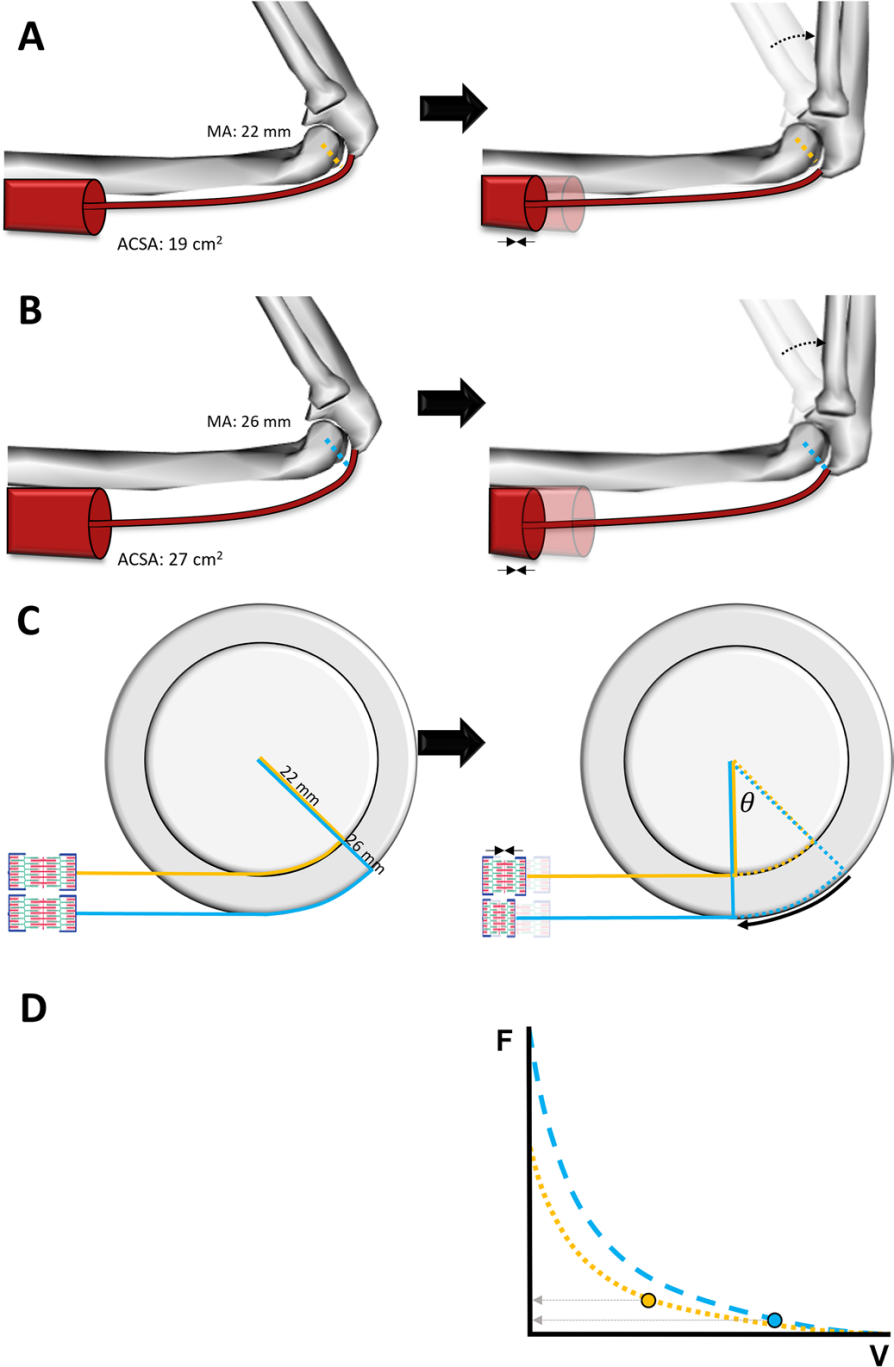
Schematic diagram of the effect of changes in moment arm due to muscle hypertrophy on muscle shortening magnitude and velocity. (**A**) Triceps brachii with an anatomical cross-sectional area (ACSA) of 19 cm^2^, with a corresponding moment arm (MA) of 22 mm (dashed orange line) in 124° of elbow flexion (left) and 90° of elbow flexion (right). (**B**) Hypertrophied triceps brachii with an anatomical cross-sectional area of 27 cm^2^, with a corresponding moment arm of 26 mm (dashed blue line) in 124° of elbow flexion (left) and 90° of elbow flexion (right). (**C**) Moment arms for the “normal” and “hypertrophied” muscles are depicted in orange and blue, respectively, on the circle and connected to a Hill-type muscle element (left). The linear relationship (ΔL = Δ $\theta$⋅ *r*) between joint angle ($\theta$) expressed in radians, moment arm length (radius, *r*) and range of muscle excursion (delta length, ΔL) dictates that the hypertrophied muscle has to shorten more (increased ΔL) to produce a given change in joint angle (Δ $\theta$) than the non-exercised muscle due to the larger internal moment arms (26 mm versus 22 mm). Similarly, the hypertrophied muscle has to shorten at a higher velocity to produce a given joint angular velocity than the muscle with a smaller moment arm. (**D**) The hypothetical operating regions on the force–velocity relationship for the “normal” and “hypertrophied” muscles are depicted in orange and blue, respectively. Importantly, the “hypertrophied” muscle operates at a higher shortening velocity, thus reducing force potential despite a higher absolute force potential due to muscle hypertrophy (assuming muscle hypertrophy does not increase fascicle length). Triceps brachii cross-sectional area and moment arms are based on [92]. Note that substantive hypertrophy may be required for this effect to occur

Although a potential detrimental effect of significant muscle hypertrophy on endurance performance is well established, it is less often considered to be detrimental to high-speed movement performance. However, hypertrophy can increase the (perpendicular) distance from a muscle’s line of action to the joint center of rotation, i.e., the muscle’s internal moment arm [10, 91–94] (Fig. 1). The training-induced increases in muscle cross-sectional area and internal moment arms increase the net joint moment produced during isometric and slow-to-moderate shortening-speed contractions (e.g., thus increasing “maximal” strength expressed as a maximal joint torque). However, larger moment arms may conversely also impair force production during higher-speed tasks by forcing the muscle to operate at faster shortening velocities [10, 95]. Specifically, the reduced muscle force production with larger moment arms during higher-speed tasks results from faster muscle–tendon shortening velocities for a given joint angular velocity (Eq. 1 and Fig. 1) [91, 95]. The subsequent increase in muscle fiber shortening velocity in turn decreases force production according to the inverse hyperbolic force–velocity relationship [96], potentially offsetting the increase in joint moment resulting from the increase in moment arm itself. As a result, peak joint power may be maintained but the joint angular velocity at which peak power occurs may be reduced.

1 $${\text{Muscle}} - {\text{tendon velocity }}\left( {{\text{m}}/{\text{s}}} \right) \, = {\text{ moment arm }}\left( {\text{m}} \right) \cdot {\text{joint angular velocity }}\left( {{\text{rad}}/{\text{s}}} \right)$$

In fact, the faster maximum joint angular velocity and slower fiber shortening velocity for a given joint angular velocity allowed by smaller internal moment arm lengths could theoretically benefit athletic performance in sports that require high-speed movement capacity. In partial support of this notion, trained sprinters have been found to have smaller plantar flexor [97, 98], but not knee extensor [99], moment arms than height-matched non-sprinters. The different associations observed for the effects of ankle and knee moment arms may reflect the approximately threefold higher power generation at the ankle than the knee during maximum speed sprinting [100], with the mean peak angular velocities at the ankle also being ~ 2–3 times faster than that at the knee (~ 1500 deg/s versus ~ 600 deg/s for the ankle and knee, respectively [100–102]). This therefore reduces the need for smaller knee moment arms as compared to the ankle. Shorter moment arms may therefore be beneficial for joints that exhibit fast angular velocities such as the ankle joint during sprinting.

In parallel, some studies also show shorter Achilles tendon or patellar moment arms to be associated with a better running economy at moderate running speeds (> 12.6 km/h) [55, 94, 103–106]. Increasing muscle size to the extent where it may increase internal moment arm lengths could thus theoretically also be detrimental to moderate-speed submaximal running performances. However, some studies reported longer Achilles tendon moment arms to be associated with better running economy [107, 108], or showed no association with running economy [109, 110], although all but one study [107] were performed at slower running speeds (< 12.6 km/h). This could imply that the ankle angular velocity at moderate running speeds is already so high that a smaller moment arm and the resulting slower muscle–tendon unit velocity may outweigh the smaller joint moment produced due to the smaller internal moment arm itself.

Nonetheless, the likelihood that hypertrophy-induced increases in internal moment arm lengths meaningfully affect high-speed movement performance may be considered small because substantial muscle hypertrophy (e.g., of the order seen in bodybuilders or powerlifters) seems to be required before a detrimental effect of a longer moment arm outweighs the muscle force production benefit. For example, Sugisaki and colleagues [93] found that a 34% increase in triceps brachii anatomical cross-sectional area was accompanied by an increase of only 6% in the internal moment arm length relative to the elbow joint, corresponding to about 3 mm for a 5-cm moment arm. Similarly, the relation between calf circumference and plantar flexor moment arm has been found to be moderate (*R*^2^ = 23% [10]), suggesting that the genetic influence or training-induced differences in muscle size are generally accompanied by only small differences in moment arm. Finally, the effects of muscle hypertrophy on moment arm may be muscle-group specific. For example, the patellar tendon moment arms of long-term (> 3 years) strength trained individuals did not differ from untrained individuals (after correcting for standing height) despite the substantially greater muscle volume of the strength-trained individuals [111]. This result can be explained by the patella (bone) thickness primarily influencing the patellar moment arm length, with the thickness of the quadriceps muscles unable to further affect the joint-to-tendon distance (although it may affect the “quadriceps tendon” moment arm). Nevertheless, the concept of hypertrophy-induced increases in muscle moment arm lengths suggests that increases in strength past some specific point may not further benefit high-speed capacity (e.g., [19]), and might thus partly explain anecdotal reports of some coaches and observations in the literature [35–37] that substantial muscle hypertrophy may compromise rather than benefit high-speed movement performance. Within this context it is important to emphasize that inertia effects of increases in mass are likely to have a more substantial effect on athletic performance than the moment arm effects discussed above. Moreover, the identification of the exact point beyond which increases in muscle mass will become detrimental also requires consideration of the specific muscles in question, the sport, discipline, and other adaptative responses as discussed further below. Due to such complexity, this optimal point remains to be fully established.

In summary, current evidence suggests that substantial hypertrophy is required for the increase in muscle moment arm to meaningfully influence (reduce) movement speed or affect running economy, and this effect may also be limited to specific joints. Strength training should therefore not be omitted for this concern, although sprint and endurance athletes may want to avoid extreme levels of hypertrophy to minimize possible detrimental effects. Such extreme levels of hypertrophy are most pronounced in response to high-volume strength training programs (e.g., > 45 sets per week per muscle group) performed to muscular failure [8, 112, 113].

#### Muscle Architecture

A muscle’s architecture strongly affects its force production and energy cost characteristics [50, 114], and may also influence injury risk [115], irrespective of the muscle’s size. Since different training modalities can induce distinct alterations in muscle architecture, it becomes important to understand how training-induced changes in muscle architecture may theoretically enhance physical performance and reduce injury risk. However, the effects of specific training modalities on muscle architecture are only rarely considered in practice. In the following section, we will discuss selected associations between muscle architecture and both performance and injury risk, respectively, and provide examples of how specific RT modalities may positively or negatively affect performance and injury risk due to their effects on muscle architecture.

##### Fascicle Length and Pennation Angle: A Trade-Off Between Speed and Endurance

**Fig. 2:**
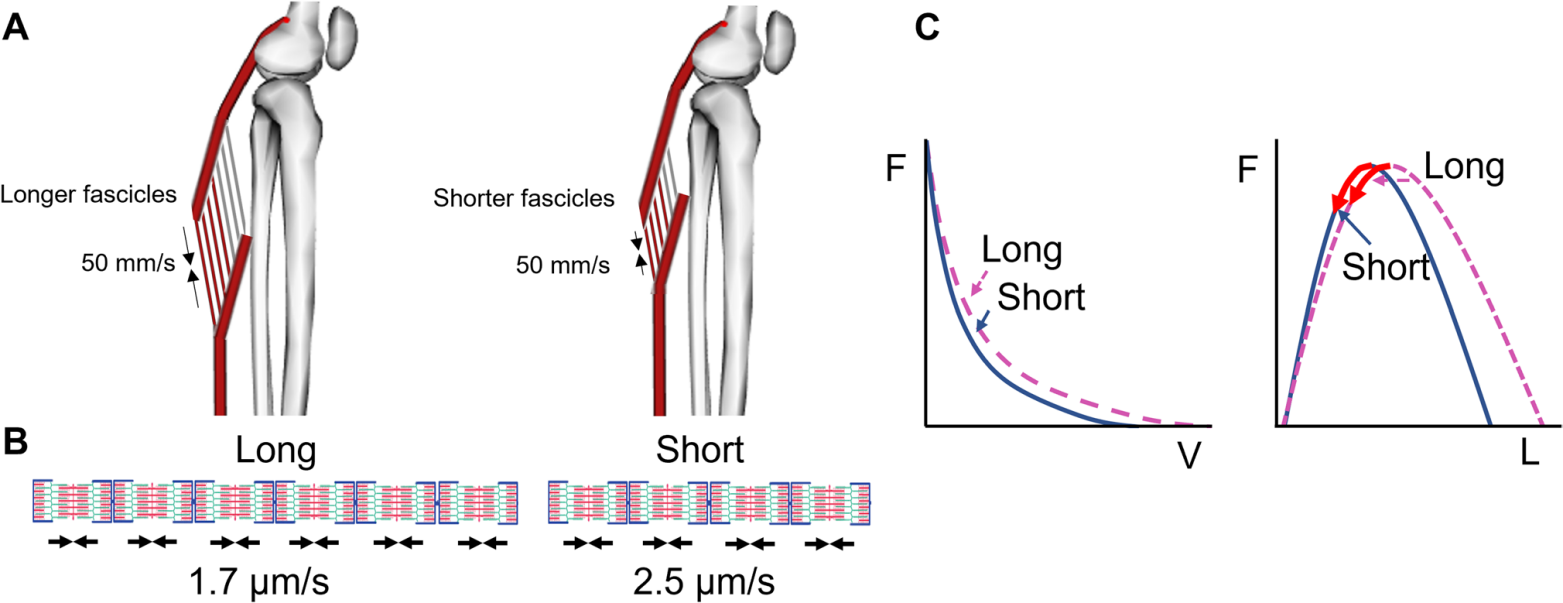
The effect of fascicle length on the sarcomere force–length velocity relationship during muscle shortening. (**A**) schematic image of a pennate muscle with relatively long fascicles (e.g., 6 cm) and therefore more sarcomeres in-series (left) and short fascicles (e.g., 4 cm) with fewer sarcomeres in-series (right). Fascicle shortening velocity is assumed to be 50 mm/s for both muscles. (**B**) the corresponding sarcomere shortening velocity is slower (e.g., 1.7 μm/s) for the longer fascicle (left) than for the short fascicle (e.g., 2.5 μm/s) due to the greater number of sarcomeres in-series. (**C**) The slower shortening velocity of the longer fascicle results in a higher force output according to the hyperbolic force–velocity relationship (left; dashed line), and also results in less shortening, which affects the force potential according to the force–length relationship (right; dashed line). Although the longer fascicle can also produce more force at a given slow shortening velocity (e.g., stance phase of running), the total active volume (summed length of all red fascicles in this schematic) is higher compared to the muscle with shorter fascicles. Therefore, although the muscle with shorter fascicles has to recruit additional fascicles to compensate for the lower force output per fascicle due to the higher shortening velocity, total active volume remains smaller if shortening velocity is low, thus favoring shorter fascicles for economy. Operating region on the force − velocity and force–length relationship is approximated based on soleus behavior during running from [64] and [153], respectively. *F* force, *V* velocity, *L* length

RT can trigger fascicle length [116] and pennation angle [39] alterations. Mechanistically, these alterations should impact performance because longer fascicles are often assumed to contain longer muscle fibers with greater in-series sarcomere number [117]. While it is still debated whether modest periods of training (e.g., weeks to months) are sufficient to promote in-series addition of sarcomeres in humans [118–120], any long-term increase in fascicle length and thereby in-series sarcomere number might benefit sprint performance because, for a given absolute fascicle shortening velocity, sarcomere shortening velocities should be lower in longer versus shorter fascicles. The slower sarcomere shortening velocity caused by sarcomere addition should then increase force production according to the force–velocity relationship. Additionally, because the sarcomeres undergo smaller length changes, they have the opportunity to operate closer to their optimum length and thus generate more force according to the force–length relationship (Fig. 2). Finally, for a given maximum sarcomere shortening velocity, longer fascicles exhibit a greater maximum fascicle shortening velocity because serial sarcomere shortening velocities are additive. The consequence is a higher maximal joint angular velocity due to the strong correlation between fascicle shortening velocity and joint angular velocity, at least in single-joint tasks [121–123]. In high-velocity, multi-joint tasks, this association is less clear due to a complex contribution of additional factors to joint angular velocity such as intermuscular coordination, tendon recoil, and pre-tension [124], as well as the more pronounced effect of muscle gearing (caused by fascicle rotation during contraction), which reduces the *fascicle* shortening velocity for a given *muscle* shortening velocity [125, 126]. Nevertheless, in support of the relevance of muscle architecture to athletic performance, longer fascicles (relative to limb length) in selected lower limb muscles have been associated with faster sprint running performance [48, 127–129], enhanced peak power output during cycling and other tasks (e.g., countermovement jumping) [130–132], while also being associated with better combined sprint and endurance performance [132]. Similarly, sprinters have been shown to exhibit longer fascicles in some lower-limb muscles than non-sprinters [98, 133].

Longer fascicles are also speculated to reduce muscle injury risk. For example, longer biceps femoris long head fascicles have been associated with reduced risk of hamstring strain injury [115, 134, 135]. One hypothesis for this association is that sarcomere length changes are smaller for a given overall muscle length change if fascicles (and thus fibers) are longer (assuming this reflects an increased number of sarcomeres in series). Since fiber strain (i.e., percent elongation) largely predicts damage magnitude during eccentric muscle injury protocols [136–138], increased microscopic damage in shorter fascicles with each length change may accumulate and thereby increase the risk of a macroscopic strain injury. Nevertheless, since muscle (e.g., hamstring, calf) strain injuries typically occur at the myotendinous junction rather than within the muscle fiber itself [139], other factors may also contribute to this association.

While longer fascicles thus appear beneficial for speed and, potentially, for (muscle strain) injury risk reduction, shorter fascicles and increased (steeper) fascicle (pennation) angles within some important locomotor muscles may benefit movement economy and hence yield positive enhancements in endurance performance. Mechanistically, shorter fascicles may benefit running economy because a smaller muscle volume needs to be recruited to produce a given force during quasi-isometric conditions (serial sarcomere number does not impact isometric force potential) [50], such as those performed by the plantar flexors during running, hopping, and bounding [64, 140]. Because the volume of active muscle mass is the product of fascicle length and the recruited cross-sectional area, muscles with shorter fascicles require a smaller recruited muscle volume for a given force requirement (see, for example, the smaller active muscle mass in the right muscle in Fig. 2) and therefore consume less energy. Additionally, shorter fibers are stiffer, which reduces the activation required to produce a given force during an eccentric muscle action, thus benefiting energy costs, and which may increase the rate of force development (RFD) [141]. In support of this concept, endurance runners tend to exhibit shorter vastus lateralis and both gastrocnemius medialis and lateralis fascicles as well as greater pennation angles than sprinters [133] or untrained individuals [133]. Further, although there are some conflicting findings [142], shorter fascicles and greater pennation angles in selected lower limb muscles have also been associated with a better running economy in trained distance runners [143] as well as in power- and endurance-trained athletes [131]. Similarly, shorter soleus (but not gastrocnemius) fascicle lengths were associated with better marathon running performance in (inter)national level runners [144]. The positive effect of pennation angle on endurance performance may be related to an increased gearing effect (i.e., increased contribution from fascicle rotation to overall muscle shortening), which can reduce fascicle shortening velocity for a given muscle shortening velocity, thereby enhancing fascicle force production [126, 145, 146]. The additional physiological cross-sectional area provided by pennation also increases the peak force production for a given volume of muscle, ensuring that less overall muscle mass needs to be recruited to produce a required force, reducing the cost of activation and thus overall energy cost [50].

In summary, the optimum fascicle length depends on the task of the muscle, with muscles producing force over a large range of joint motion and at high shortening speeds potentially benefiting from longer fascicles (but without large angulation) while muscles that remain quasi-isometric potentially benefiting from shorter fascicles at greater angles to reduce energy cost.

Due to the functional importance of muscle architecture, it is necessary to consider (a) the likelihood of inducing meaningful alterations in muscle architecture using a given exercise training program, and (b) how selected training modalities might impact muscle architecture. It is well known that RT can alter muscle architecture [39, 116, 147], even when used as part of a concurrent training program [147, 148] or in elite athletes who perform a high volume of other training elements [148]. For example, heavy (slow-velocity) RT in addition to sprint training may decrease fascicle length while (higher-velocity) ballistic or plyometric training may increase length [147, 148]. Yet the impact of architectural changes on performance may be smaller in a concurrent training setting than a stand-alone program [147], likely due to opposing stimuli and/or short restitution time between successive training sessions. Moreover, the relationship between changes in muscle architecture measured at rest and the muscle’s function during performance has only rarely been studied and requires further scrutiny. Nevertheless, the current evidence suggests that practitioners should consider the impact of specific (resistance) training modalities on muscle architecture and thus on athletic performance and injury risk. For example, large volumes of training at short muscle–tendon unit lengths (e.g., cycling during off-season in sprint/team sport athletes [149]) may lead to shorter fascicle lengths [150–152] and hence reduce performance and increase muscle strain injury risk in athletes who engage in high-speed running when returning to regular training and competition. Such architectural adaptations may offer an additional explanation as to why some coaches and athletes do not necessarily report beneficial effects of RT on sports (e.g., sprint) performance.

#### Tendon Stiffness: A Trade-Off Between Speed and Strength Performances

Tendons play an important role in optimizing movement performance and reducing injury risk. Tendon lengthening during activities in which external forces rapidly stretch the muscle–tendon unit, for example, reduces the magnitude and velocity of muscle stretch and thus may help prevent strain-induced damage to muscle fibers [154]. Tendon elongation during force production can also allow muscles to work at more favorable points on the force–length–velocity relationship by minimizing whole-muscle length change, which optimizes force potential and movement economy [155, 156]. Finally, tendons can store and then rapidly return the energy of muscle work (or gravity) as elastic strain energy, thereby increasing overall muscle–tendon unit power output [156]; that is, tendons may act as power amplifiers. The mechanical properties of tendons must however be matched (i.e., tuned) to their function for maximum effectiveness [157–160].

**Fig. 3:**
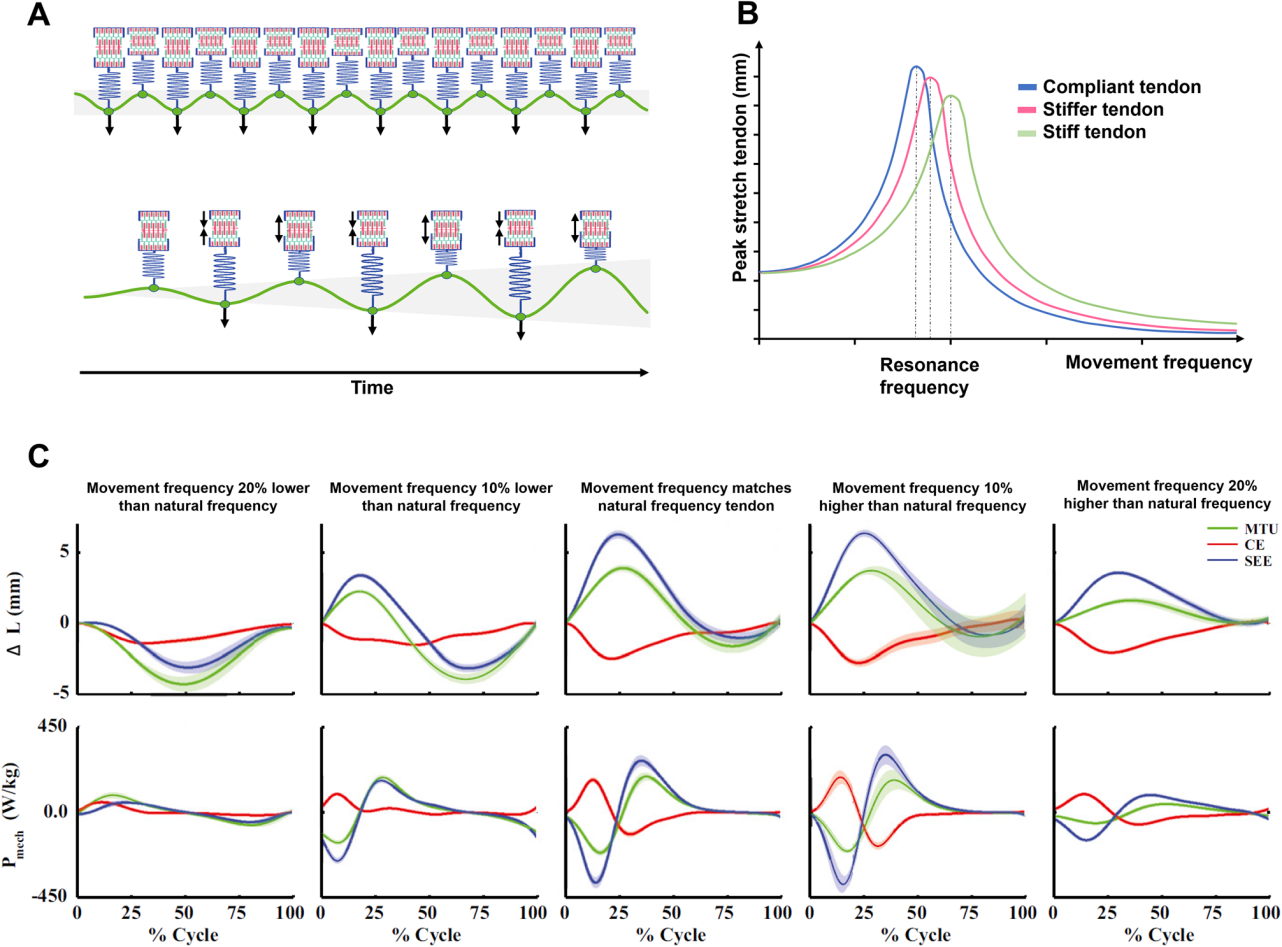
The effect of the tendon’s natural oscillation frequency on tendon stretch amplitude and mechanical power storage and release. (**A**) Schematic illustration of a Hill-type muscle model working at two different movement frequencies. In the top illustration, the movement frequency depicted by the green line (muscle–tendon unit length) is high relative to the tendon’s natural frequency. As a result, the tendon is stretched only a small amount and does not fully recoil before the subsequent force increase stretches the tendon. In the bottom illustration, the movement frequency is slower and matches the tendon’s natural frequency. Therefore, resonance (i.e., large amplitude oscillation) is achieved over time and energy is added to the system because internal muscle shortening is effectively stored and released as elastic energy by the tendon. (**B**) Schematic illustration of the tendon peak stretch amplitude as a function of movement frequency for tendons of different stiffness. At an optimum movement frequency, the tendon’s natural oscillation frequency is in resonance with the movement frequency. This increases peak tendon stretch amplitude relative to both higher and lower frequencies. As higher tendon stretch stores more elastic energy for a given tendon stiffness, the larger tendon stretch at resonant frequency can increase muscle–tendon power output. The frequency at which resonance occurs increases with tendon stiffness. (**C**) Ex vivo data depicting the effect of changes in movement frequency for a given tendon stiffness on muscle–tendon unit length changes and mechanical power production, adapted with permission from Robertson and Sawicki [158]. The center plot depicts resonance between the tendon’s oscillation frequency and movement frequency, which causes maximum tendon stretch (blue line in upper panel) and hence the highest muscle–tendon power output (green line in lower panel). The left plots depict movement frequencies that are lower than the tendon’s natural frequency. At low movement frequencies relative to the tendon’s natural frequency, the tendon acts like a rigid material and the muscle fibers have to produce work by shortening, with less benefit from tendon elastic energy return. Conversely, right panel plots depict movement frequencies higher than the tendon’s natural frequency, resulting in work performed by the tendon being absorbed by the muscle and vice versa, leading to a net reduction in musculotendinous mechanical power production [158, 164]. The tendon’s natural (resonance) frequency in this experiment was 2.34 Hz, while − 20% and − 10% slower movement frequencies were obtained at 1.87 and 2.11 Hz, respectively, and + 10% and + 20% faster movement frequencies were examined at 2.57 and 2.81 Hz, respectively

Theoretically, movements requiring large ranges of motion or relatively low muscle forces (e.g., swimming) may benefit from more compliant tendons because compliant tendons will stretch further for a given load, and their energy storage can be substantial given the large joint ranges of motion allowed by the movement task. Specifically, energy storage increases approximately to the square of the elongation distance, in accordance with Hooke’s Law (stored elastic energy = ½*kx*^2^, where *k* is tendon stiffness and *x* tendon elongation). Relative to a stiffer tendon, more compliant tendons will stretch further and thus store more elastic energy for a given muscle force. In addition, more compliant (i.e., less stiff) tendon properties may also optimize energy store-release efficiency at slower movement frequencies (resulting from larger ranges of motion) since the lower natural oscillation frequency of compliant tendons (Eq. 2) ensures greater power amplification and reduced muscle fiber energy cost at such frequencies [158, 161–164]. Specifically, when the tendon’s natural frequency matches the frequency of the movement (i.e., they act in resonance), the loss of energy is minimized [165, 166], the tendon stretches and recoils further, and the muscle shortens less and at a slower velocity (Fig. 3). Ipso facto, muscle work increases as the muscle and tendon length changes occur more out of phase, with the muscle lengthening as the tendon shortens and vice versa. Work performed by one element is therefore absorbed by the other, thus reducing overall mechanical power production and potentially leading to a reduced movement economy.

2 $$\text{Natural oscillation frequency in tendon }\left(\text{Hz}\right)=\frac{\sqrt{\frac{k}{m}} }{2\pi }$$

where *k* is the stiffness of the tendon (in N/mm), and *m* is the inertial mass (in N) carried by the tendon. Increasing tendon stiffness will, therefore, lead to increases in the natural resonance frequency, while increased mass will reduce this.

In support of the concept that movements requiring large ranges of motion may benefit from more compliant tendons, a lower patellar–tendon stiffness has been associated with better sprint running performance [167, 168], possibly because the knee range of motion is large and patellar tendon forces are low relative to the ankle-Achilles tendon (force ratio ~ 0.67 [169]). Further support for benefits of compliant tendons in movements requiring large ranges of motion or relatively low muscle forces is provided by modelling studies that show a more compliant tendon in several lower limb muscles (10–15% strain at maximum isometric muscle force) leads to increased squat jump height than a more rigid tendon (1 or 4% strain) [170]. Note, however, that further increases in compliance may be less beneficial to jump height because the muscles shorten further and faster against the compliant tendon, thus forcing the muscle to function at a suboptimal part of force–velocity and force–length relationships. At first glance, experimental studies do not unanimously support this concept as Achilles and patellar tendon stiffness are not always correlated with countermovement or squat jump performances [171] and some studies even report greater stiffness to be associated with better jump performance [172, 173]. Nonetheless, numerous factors may influence overall vertical jumping performances (including maximal lower limb muscle power, muscle fiber type proportion, technique, etc.), so it may be argued that the countermovement-to-squat jump ratio may better assess the influence of tendon stiffness on jump performance as it minimizes the effects of inter-individual differences in muscle power capacity, technical competence and other factors because they are common to both jump types, whereas elastic energy storage-recovery is supposed to be (slightly) more influential in the CMJ [174]. Using this approach, both modelling [157] and empirical evidence [172, 175–177] suggests that those with more compliant tendons have a higher countermovement-to-squat jump ratio. Finally, athletes who frequently perform countermovement jumps (volleyball and basketball athletes) have been shown to exhibit a lower Young’s modulus in the patella tendon than untrained individuals [178].

In contrast, relatively stiff tendons may be beneficial in situations in which high external forces rapidly stretch the tendon and when the joint range of motion (and thus muscle–tendon length change) is limited. In this case, stiff tendons will store more energy for a given (restricted) length change and then recoil at a greater rate (due to a higher natural oscillation frequency), hence resulting in a higher muscle–tendon unit power. For example, ankle dorsiflexion during the stance phase of running is restricted to approximately 20° and tendon forces are high (i.e., up to 8× body weight), so one might expect a relatively stiff tendon to provide a greater recoil power and reach a higher resonance frequency coinciding with the fast stride rate of sprinters. In partial support of this notion, sprinters have been shown to have stiffer Achilles tendons than untrained individuals [179], although this is not observed in all studies [180]. A potential reason for these conflicting findings is that faster sprinters may also have longer tendons [128], with longer tendons being more compliant if tendon cross-sectional area and material properties are similar. The longer tendon length could reduce stiffness, while the higher Young’s modulus could increase tissue-specific stiffness, thus partially masking the effects of stiffer material properties on performance outcomes. The muscle-specific optimal stiffness (patellar versus Achilles) may also explain why modeling studies that uniformly increase the stiffness for all muscle–tendon complexes within the body do not find clear effects of increased/decreased tendon stiffness on sprint performance [48].

Although a stiff Achilles tendon could theoretically be detrimental to running economy by reducing elastic energy storage and release in the tendon [160, 181], both cross-sectional [182] and training [183, 184] studies have shown stiffer Achilles tendons to be associated with better running economy. This results from a stiffer Achilles tendon stretching less under the high forces produced during foot–ground impact than a more compliant tendon, reducing the requirement for muscle shortening and therefore reducing the shortening velocities of the active muscle fibers, thereby reducing muscle energy cost via optimization of the force–length–velocity relation [155]. Note that the calf muscle fibers are not stretched during ground contact (under normal conditions from 3 to 8.4 m/s) even though the muscle–tendon unit itself undergoes a stretch-shorten cycle [185]—efficiency is therefore optimized when the muscle works quasi-isometrically rather than shortening substantially and rapidly.

In addition to its role in optimizing running and jumping performances, tendon stiffness may also speculatively play an important role in the risk of musculoskeletal injury. Mechanistically, strain (relative lengthening) of a compliant tendon will be greater for a given force. A greater strain renders the tendon more susceptible to incidents of collagen fibril micro-rupture, which, without sufficient time for repair and adaptation, may eventually accumulate into overuse injury (e.g., tendinopathy) or tendon rupture [186–188]. It is therefore important that tendon stiffness is matched to the strength of the muscle to prevent excessive tendon strain [189]. While stiffer tendons may therefore be beneficial to reduce tendon strain and thus reduce tendon injury risk, modelling studies show that a stiff tendon can increase the magnitude of muscle fiber stretch (and thus increase the risk of muscle strain injury) during forceful muscle–tendon unit lengthening such as in eccentric actions or maximal stretch–shortening cycles, e.g., the hamstrings during the swing phase of high-speed running [190]. With stiffer tendons, the muscle may therefore theoretically become more susceptible to strain injury or damage.

Given the potential impact of tendon stiffness on both performance and injury risk, it is important to consider (a) the likelihood of inducing meaningful alterations in tendon stiffness using an exercise training program, and (b) how selected training modalities might impact tendon stiffness. In this regard, numerous studies have shown that heavy resistance training leads to substantial increases in tendon stiffness [3, 191], with less consistent effects of moderate intensity resistance training or plyometric training programs [3]. However, the effects of training-induced changes in tendon stiffness on in vivo muscle–tendon behavior are less clear [192, 193]. The lack of clear associations between alterations in tendon stiffness and in vivo muscle function may partly reflect the modulation of muscle–tendon stiffness by various feedforward and feedback neural control strategies [160, 194–196] that may adapt muscle behavior to alterations in tendon stiffness. While some effects of tendon stiffness change on muscle behavior may therefore be compensated for by alterations in muscle activation patterns, the lower levels of muscle activation with higher tendon stiffness may per se reduce energy cost during submaximal tasks (e.g., [183, 184]), or allow increased power output during maximal effort tasks. Although it is likely that other adaptations (e.g., extracellular matrix mechanical properties) may mask any potential negative effects of RT on tendon properties, it may for the reasons discussed above be important to understand the range of motion and forces acting on a tendon to determine the most appropriate training mix. For example, for tasks (or specific muscle-groups within tasks) in which the time available for force production is short but forces are high, heavy resistance training may benefit both performance [183, 184] and reduce injury risk [197, 198]. Given that there is no current evidence that greater Achilles tendon compliance benefits either sprint or endurance running performance, there appears to be no grounds to completely remove RT from athlete training programs for this reason. However, given that a lower patellar tendon stiffness may be beneficial for some tasks, some consideration of knee extensor (e.g., squat) training intensities and volumes may be warranted.

### Adaptations in Muscle Fiber and Myofiber Morphology

The inter- and intrafiber (i.e., microscopic) structure of a muscle also strongly influences maximum muscle force production, the rate of force production, and the energy cost of force generation, independent of its macroscopic architecture. Additionally, the microscopic structure also influences injury risk. Similar to changes in macroscopic muscle morphology and architecture, different training modalities can induce distinct alterations in microscopic muscle structure, and understanding which structures are targeted by specific interventions may help to maximize physical performance and reduce injury risk.

#### Muscle Fiber Type: A Trade-Off Between Speed and Endurance

Skeletal muscles consist of different fiber types, which vary in their contractile properties although also showing considerable functional overlap, especially in response to training [199–202]. The myosin heavy chain isoform composition is often used to classify fiber types, although other methods (e.g., histochemical ATPase staining, etc.) have also been used [203, 204]. Based on such analysis, human muscle fibers are typically classified into three main types: type I, IIa and IIx. However, hybrid fibers co-expressing multiple myosin isoforms also exist and there can be structural and functional differences even within the same fiber type [75, 205–207]. Nevertheless, type I fibers generally demonstrate a slower shortening velocity than type II fibers but are more fatigue resistant, require less energy for force production at submaximal shortening speeds, and may be less susceptible to muscle damage (at least in studies using electrical muscle stimulation) [45, 206, 208–211]. Conversely, type IIx fibers are the fastest fibers but are also highly fatigable [205–207, 212], which may increase injury risk independently (e.g. [213]). Type IIa fibers exhibit properties that are intermediate between type I and type IIx fibers. As such, type I fibers are generally more suited to endurance activities whereas type IIa, and particularly type IIx, fibers are better suited to rapid force production. In support of this concept, endurance athletes generally exhibit a higher proportion of type I fibers in lower limb muscles whereas sprinters and/or power athletes have a higher proportion of type II(x) fibers [212, 214–219].

Anecdotally, coaches engaged in endurance sports sometimes discourage their athletes from engaging in RT because they believe it may trigger a shift from type I to II fibers, which in turn could be detrimental to movement economy and overall fatigue resistance. Conversely, some sprint coaches argue that RT shifts type IIx to type IIa fibers or type II to type I fibers, both of which could be detrimental to sprint performance. The paragraphs below briefly review the evidence for these assertions.

Firstly, numerous studies have reported no shifts from type I to type II fibers with heavy RT alone [39, 205, 208, 220] or in a concurrent program [75, 221]. Indeed, any decrease in type I fiber content is often non-significant and relatively small (< 1% points average change) both in isolated (single mode) and combined strength and endurance programs when compared with endurance training alone [75, 76, 222]. Heavy-load RT should therefore not be omitted from an endurance program for this reason. Yet some, but not all [202], studies show that low-load high-velocity RT alone may in some conditions lead to shifts from type I to type IIa fibers (~ 10% points average change) [223, 224], although type IIx content often also decreases. Mechanistically, early animal studies suggested that shifts in fiber type can be attributed to the innervation of the motor unit (i.e., frequency of neural stimulation), with an important role for intra-myocellular signaling pathways (for a brief review see Plotkin and colleagues [203]). Endurance athletes may therefore take care when prioritizing high-velocity training (with longer rest periods) as this may shift type I towards type II fibers, although this has so far not been observed in elite endurance athletes in concurrent training programs [75]. Notably, for power athletes such a shift may be beneficial.

Conversely, prolonged endurance training can shift pure type IIa or hybrid IIa/IIx to hybrid I/IIa fibers or pure type I fibers. For example, 13–18 weeks of endurance training in previously untrained or recreationally trained individuals has been reported to increase type I fiber content in various lower limb muscles by 5–15% points [225–229]. Similarly, both isolated heavy RT or a combination of heavy RT and endurance training consistently appears to result in a shift from type IIx and type IIx/IIa hybrid fibers to pure type IIa fibers [75, 76, 205, 207, 208, 220, 221, 230, 231]. For endurance athletes, such a shift is beneficial as type IIa fibers are more fatigue resistant and more economical than IIx fibers, the latter due to their faster rate of cross-bridge cycling and higher activation costs [50, 75, 205]. Indeed, Vikmoen and co-workers [76] showed a moderate association (*r* =  − 0.63) between the decrease in type IIx fiber proportion and increase in power output during a 40-min all-out cycling test following 11 weeks of concurrent resistance and endurance (cycling) training in well-trained female cyclists. Conversely for sprint athletes, such a shift (type IIx → IIa) may be detrimental as type IIx fibers show the highest (association with) RFD [205, 207, 212]. Indeed, reductions in the proportion of type IIx fibers have been associated with decreases in RFD in the early phase (0–50 ms) of rising muscle force [5, 232], which could be detrimental to high-speed performances in conditions with restricted time (≤ 50–100 ms) to produce propulsive impulse. Decreases in type IIx fiber proportions occur in particular when the training volume is high [5, 205] and when training closer to failure [4, 205], i.e., when muscle fatigue is substantial. In contrast, low-volume power (i.e. higher-speed, lighter-load) or heavy resistance training has been reported to (largely) conserve type IIx fibers [4, 5, 233–235] and may therefore be better suited to improve RFD and thus speed performances in sprinters. While heavy RT is therefore unlikely to be detrimental—and in fact likely beneficial—to muscle fiber typology for endurance or team sport athletes, it may be detrimental in sports that require single, all-out explosive efforts such as track and field sprinting if performed in large volumes as this may reduce the type II fiber content at the expense of type I fibers. This effect may therefore also partly explain the anecdotal observation by sprint coaches that high volumes of RT may sometimes (at least transiently) impair performance in well-trained sprinters. Importantly, there is also some evidence of a rebound in type IIx fibers during a tapering period after periods of heavy RT, which could benefit sprint performances [205, 232], although this needs further scientific exploration. It is also important to acknowledge that team sport athletes often need to perform multiple sprints, and these individuals may benefit from a conversion of type IIx into type IIa fibers to improve repeated sprint performance, based on the greater fatigue resistance of type IIa fibers (although data explicitly testing this hypothesis are lacking).

#### Mitochondrial Adaptations and Capillarization: A Trade-Off Between Strength and Endurance

Mitochondria are intracellular organelles that play a critical role in energy generation for muscle contraction [236]. Indeed, mitochondrial oxidative capacity is strongly correlated with body mass-specific peak oxygen uptake [214]. Since peak oxygen uptake is an important determinant of endurance performance [214], maximizing mitochondrial oxidative capacity is important to enhance endurance performance.

RT has often been reported to positively impact mitochondrial biogenesis and respiratory function (and thus oxidative capacity) in untrained individuals [236, 237], which can therefore benefit endurance performance. Similarly, some studies show a higher mitochondrial density following RT [238]. However, other studies report no beneficial effects of RT on mitochondrial function, or even report negative effects [239, 240]. One study for example reported that seven weeks of RT prior to endurance training blunted mitochondrial adaptations to the endurance training [240]. The reasons for these conflicting findings may be related to the exact RT program and training status of the participants. Further, substantial fiber hypertrophy following RT can also negatively impact mitochondrial density [241–243], which impairs ATP diffusion to muscle fibers and can in turn impair endurance performance at the expense of increasing muscle strength. Indeed, in relatively untrained individuals—who experience larger fiber growth than better trained individuals—muscle fiber hypertrophy following concurrent training has been shown to attenuate the increase in maximum oxygen consumption for this reason [89]. In further support of this concept, several studies in both animals and humans have reported inverse correlations between muscle fiber cross-sectional area and mitochondrial oxidative capacity [132, 244], and in turn also between muscle fiber cross-sectional area and endurance performance or the lactate threshold [214]. While athletes can partly compensate for increased intracellular diffusion distances resulting from muscle fiber hypertrophy by increasing capillary-to-fiber ratio, capillary density (cap/mm^2^), and hemoglobin concentrations, amongst others [75, 132, 214], there are likely limits to this compensation. Indeed, while RT has been reported to increase the number of capillaries per fiber in previously untrained individuals [245–248], it did not further increase the high number of capillaries per fiber observed at baseline in young National Team cyclists (6–7 cap/fiber) [75], hinting at an upper limit for RT to positively impact this parameter.

Collectively, current evidence suggests that there may be a trade-off between maximizing strength by increasing muscle fiber cross-sectional area and maximizing endurance capacity by maximizing mitochondrial number and capillary density with minimal fiber hypertrophy. It is important to note, however, that a small amount of fiber hypertrophy, in particular in type I fibers, is likely beneficial to endurance performance [214] because the increased contractile strength of type I fibers delays the recruitment of the less economical type II fibers, which may partly explain why concurrent training sometimes improves movement economy (only) when measured under fatigued conditions [249]. Further, neither concurrent training nor RT without excessive hypertrophy typically reduce mitochondrial density or muscle fiber capillarization [75, 80, 221]. This likely results from large volumes of endurance training interfering with muscle fiber hypertrophy and thereby minimizing potentially detrimental effects of RT on mitochondrial density and capillarization, in particular in running-based sports. While large RT volumes might therefore be avoided to temper increases in fiber cross-sectional area [5], in particular of type II fibers [39, 205, 243], smaller volumes of RT are unlikely to stimulate maladaptive changes in mitochondrial density or muscle fiber capillarization and rather seem to result in positive adaptations such as improvements in maximal muscle strength [75–77, 81], RFD [75] and maximal muscle power output [76, 77, 81], which collectively may improve movement economy [76, 250] and running or cycling time trial performances [75–77, 81]. These findings therefore suggest that concerns regarding maladaptive adaptations in mitochondrial density or muscle fiber capillarization due to RT in endurance athletes are unjustified as long as fiber hypertrophy is not substantive.

#### Myofilament Density and Structure: A Trade-Off Between Strength and Speed

**Fig. 4:**
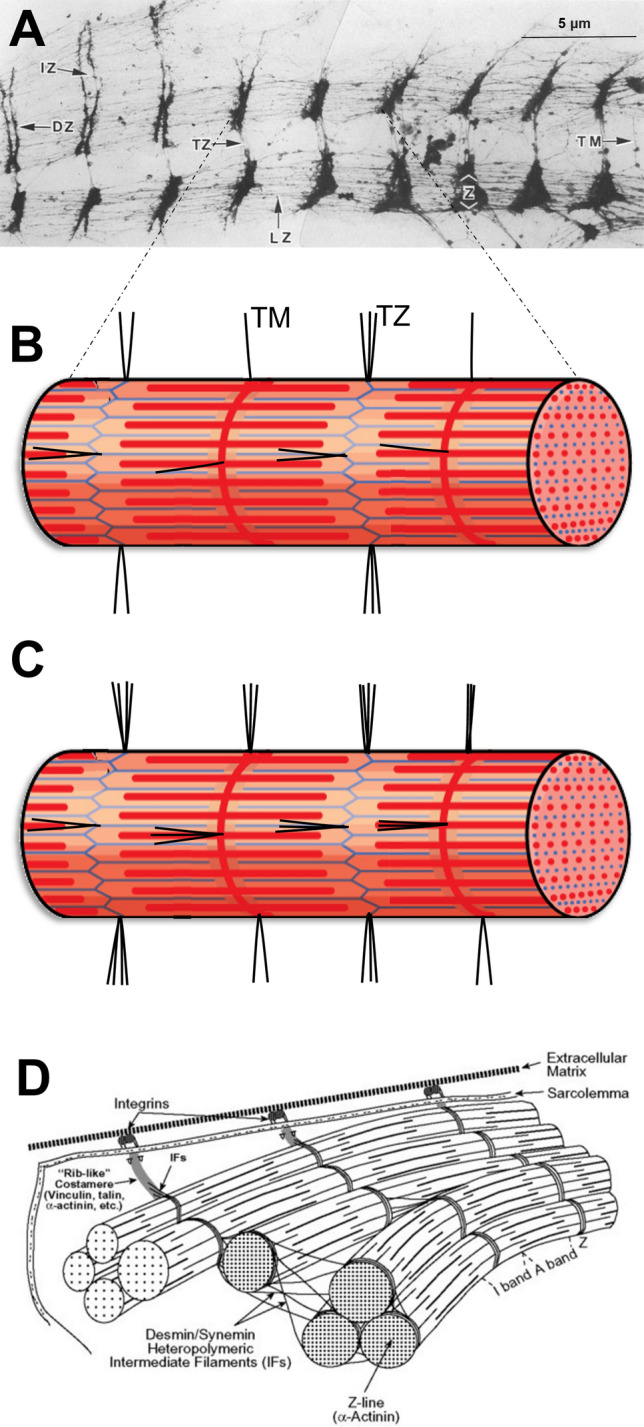
(**A**) Electron microscopic image of rabbit skeletal muscle showing the transverse intermediate filaments. TZ, transverse filaments connecting Z-bands to Z-bands; TM, transverse filaments connecting M-line to M-line; LZ, longitudinal filaments connecting Z-bands to Z-bands. (**B**) schematic of these intermediate filaments in a relatively untrained individual. (**C**) An increase in the number or thickness of intermediate filaments may be observed after a period of heavy-load resistance training. (**D**) schematic of the three-dimensional structure of intermediate filaments (IFs). Microscopic image in panel A is adapted from Wang and Ramirez-Mitchell [265] with permission, whereas the schematic in panel **D** is adapted from Robson et al. [266] with permission. Human data supporting the existence of these intermediate filaments are also available (e.g., [267]), with training studies also showing that the protein content of intermediate filaments (e.g., desmin) can increase in response to RT [220]

The properties of individual fibers containing the same myosin heavy chain isoform (i.e., same fiber type) can also change with training and thus impact performance and potentially influence injury risk. For example, sarcomeres transfer force in both lateral and longitudinal directions [251–253] and it has been proposed that heavy RT can increase the magnitude of intracellular lateral force transmission by increasing and enforcing the lateral attachments between intermediate sarcomeres and the extracellular matrix [230, 254]; Fig. 4. This could theoretically increase specific tension and hence enhance maximum force capacity but may also reduce maximum shortening velocity by decreasing the effective muscle fiber length. Although some indirect data support this effect [230], more research is required to substantiate the claim. Nevertheless, this effect could offer an additional explanation for the anecdotal observations (and observations in some studies, e.g., [19, 35]) that increasing maximum strength and muscle mass does not necessarily improve maximal movement speed.

A similar trade-off between strength and speed may occur within single sarcomeres. Specifically, an increase in the myofilament packing density (i.e., reduced lattice spacing) within a sarcomere can increase the maximum fiber force but may simultaneously decrease the maximum unloaded shortening velocity according to both animal [255, 256] and human experiments [257], although the latter is limited to bed rest conditions. This effect has been attributed to a smaller actin–myosin distance, which increases the probability of cross-bridge formation, in turn increasing force production. However, it also slows the rate of cross-bridge detachment, thus reducing cross-bridge cycling rate and hence reducing fiber shortening velocity [255]. It can therefore be hypothesized that while increases in contractile filament density following RT may benefit maximal force production, this may at the same time compromise maximum shortening velocity, although further verification of this effect is needed in humans. In line with this hypothesis, some studies report trends for simultaneous increases in fiber force normalized to cross-sectional area and decreases in maximum unloaded fiber shortening velocity following RT [258]. Likewise, normalized fiber force is higher whereas maximum unloaded fiber shortening velocity is lower in bodybuilders than untrained controls [259]. However, among subjects demonstrating relatively minimal muscle hypertrophy, neither normalized fiber force nor maximum fiber shortening velocity seem to differ from untrained individuals [260]. Finally, recent findings also indicate a higher myosin density in the biceps brachii muscle of long-term RT individuals (demonstrating 70% greater anatomical biceps cross-sectional area) compared with untrained individuals [41], although single fiber force and shortening velocity were not assessed.

These findings collectively suggest that large amounts of fiber hypertrophy may increase absolute and normalized fiber force at the expense of fiber shortening velocity, but this may not occur with smaller magnitudes of hypertrophy. Further, in contrast to the decreases in fiber shortening velocity observed in some [255–257] (but not all [258]) RT studies, speed-oriented training may increase the maximum fiber shortening velocity [202] and peak power production [261] without substantially altering maximal specific (normalized to cross-sectional area) fiber force [202, 261]. Thus, while some indirect findings support a trade-off between strength and speed at the myofiber level, the mechanisms underpinning this effect and its overall impact remain unclear. For example, while some studies do indeed detect an increase in myofilament packing density following RT [41, 262], other studies report no change [263] or show a decrease in packing density [264], with the latter likely resulting from sarcoplasmic hypertrophy [264]. Moreover, other researchers have argued that the alterations in intrinsic contractile properties primarily reflect changes within the myosin filaments themselves [259], although the effect of these adaptations on sports performance requires more research.

### Intramuscular Coordination: A Trade-Off Between Speed and Muscle Strength or Endurance

Intramuscular coordination refers to the coordinated recruitment of motor units within a single muscle, and depends primarily on the recruitment threshold of motor units and the rates at which motor neurons discharge action potentials (rate coding) [268, 269].

Alterations in intramuscular coordination have the potential to influence both sprint and endurance performances. For example, rate coding is strongly associated with the rate of force development during fixed-end (isometric at the muscle-tendon level) contractions [268]. As such, during tasks where fast force production is required (e.g., sprint start), there is great interest in improving the maximal discharge rates of the motor units innervating the prime mover muscles, in turn increasing rapid force capacity (rate of force development; RFD). A large number of studies have shown that heavy-load RT can improve RFD in both isometric [270–272] and dynamic contraction conditions (although with less pronounced effects versus isometric conditions) [273, 274], yet not all studies have reported beneficial effects of heavy-load RT on RFD [2, 275]. Del Vecchio and colleagues [2] recently showed that the absence of changes in RFD after isometric strength training, in their case, could be explained by a lack of adaptations in maximal motor unit discharge rates, suggesting insufficient adaptative plasticity at the neural level with their training protocol. Interestingly, in their study, the training protocol consisted of combined isometric strength training and rapid muscle contractions (“explosive training”), suggesting that short-term strength training may even inhibit the increase in rate of force development that is observed during explosive training alone. Such an observation may partly explain why some practitioners do not report beneficial effects of heavy-load RT on performance in explosive movement tasks.

A similar specificity of adaptations is observed in some studies comparing the outcome of contrasting strength and endurance training programs. For example, Vila-Chã and co-workers [276] showed that 6 weeks of strength training led to increased motor unit discharge rates during submaximal isometric contractions, while endurance training decreased motor unit discharge rates. Speculatively, this could reflect a trade-off between optimizing movement economy (i.e., minimizing the energy cost associated with increased motor unit discharge rates [277, 278]) versus muscle strength. Similarly, muscle activation at the onset of a rapid isometric explosive contraction was impaired by concurrent strength and endurance training as compared with strength training alone [279]. Collectively, these findings suggest that coaches should take care when implementing large volumes of heavy-load RT or endurance training with the aim of optimizing RFD or when implementing large volumes of explosive-type RT, i.e., using rapid contractions (e.g., ballistic exercises) when at the same time intending to optimize movement economy. Importantly, for endurance athletes, any negative effects with smaller volumes of training are however unlikely to occur based on the current body of evidence as most studies have rather reported beneficial effects of concurrent training involving explosive-type heavy-load RT in various athlete groups, including moderately to highly trained middle- and long-distance runners [32, 280–282].

### Intermuscular Coordination: A Trade-Off Between Individual Muscle Strength and Integrated Performance

Intermuscular coordination can be defined as the timing with which synergist muscles are activated and deactivated in relation to each other and their relative activation magnitudes. Mechanistically, intermuscular coordination is important for (a) optimizing the resultant force in both magnitude and direction, which increases the work done in the direction of body center of mass travel and thus optimizes both speed and efficiency (economy), (b) maintaining posture to allow the limbs to work off a more stable base of support (i.e., they do not store or lose/release energy that should have been transferred along the kinetic chain), (c) allowing limbs to move at optimum points in the movement task to make best use of Coriolis and centrifugal forces to accelerate limbs or an external object, and (d) optimizing energy transport from proximal to distal joints by means of biarticular muscles, which in turn affects the total power output [158, 283].

**Fig. 5:**
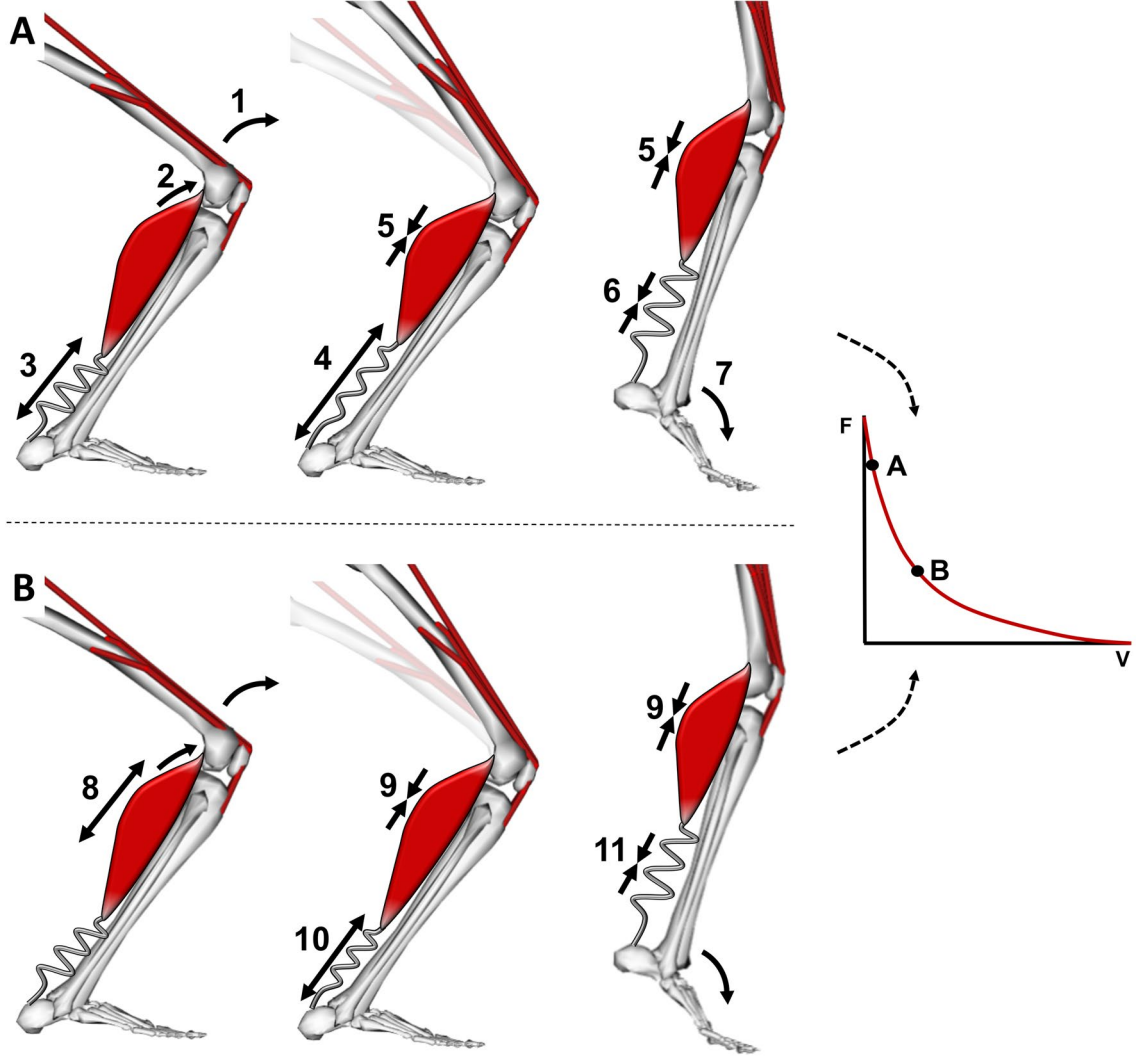
The effect of intermuscular coordination on energy transport during vertical jumping. Top (**A**): An example with optimal intermuscular coordination whereby knee extension due to the strong knee extensors (1) results in lengthening of the bi-articular gastrocnemii muscle–tendon unit (2). If the gastrocnemii are activated with appropriate timing, they will be sufficiently stiff to resist lengthening so that knee extension will primarily stretch the Achilles tendon and thereby store elastic energy (3 and 4). The storage of elastic energy in the Achilles tendon also allows the gastrocnemii to shorten at a relatively slow velocity against the increasing resistance of the tendon (5), thus allowing them to operate on a more favorable portion of the force − length − velocity relationship (see F–v relationship on right side of the figure). As the knee extensors complete their action, the gastrocnemii muscle shortening velocities increase to assist with plantar flexion, thus reducing force, and allowing recoil of the Achilles tendon (6), which contributes energy (work) to rotate the calcaneus and contributes to ankle plantar flexion (7). In this way, work from proximal muscles is transferred to more distal joints, with the recoil of tendinous tissues increasing the power output beyond that of the muscle unit itself. In contrast, in the bottom (**B**) example, the gastrocnemii are activated too late, so knee extension results in lengthening of the gastrocnemii (8). Therefore, the muscles cannot transfer the energy of the work done by proximal muscles into the Achilles tendon, meaning that less energy is available to enhance power output later in the takeoff phase. Rather, the gastrocnemii fibers must shorten more and at a higher velocity (9, and point B in the F-v relationship on the right side of the figure), which will store less energy in the Achilles tendon (compare 10 versus 4), and thus result in a less powerful recoil (11). As a result, muscle–tendon shortening velocity is lower, reducing total work and power. This will reduce jump height as the peak power of the ankle plantar flexors in isolation is ~ 200 W, yet plantar flexor peak power reaches 2000–4000 W in a vertical jump due to energy transport via biarticular muscle–tendon units crossing the two joints [284, 285, 286]

With optimal intermuscular coordination, the work done (i.e., force generated over a certain shortening distance) by (strong) muscles at proximal joints can be transported distally to the knee and ankle, with part of the work temporarily stored as elastic energy in tendinous tissues at the ankle joint and then released rapidly at high power to accelerate the body center of mass. The power generated by ankle extensors in isolation is, for example, ~ 200 W, while 2000–4000 W is produced in a maximal vertical countermovement jump, partly as a result of the energy transported to the ankle and partly due to the amplification of power by the Achilles tendon and foot arches [284, 285]. With suboptimal intermuscular coordination, muscle–tendon work is not effectively transferred or stored, and instead is transformed into segmental rotational energy that does not contribute to displacement of the center of mass or external object [63, 286, 287]. Figure 5 illustrates this concept for the vertical jump, although a similar concept applies to the stance phase of (high-speed) running [286, 288–290] and other high-speed movements (e.g., throwing/hitting). A reduced energy transport means that more work needs to be done by the more distal muscles, which may decrease both movement economy and maximal speed capacity.

In support of the relevance of intermuscular coordination to speed performance, both simulation [283, 287] and experimental studies [291] have shown that suboptimal intermuscular coordination can impair vertical jump performance. For example, superior jumpers have been shown to exhibit a later onset of ankle extension, which results in a slower gastrocnemius medialis fascicle velocity and higher Achilles tendon shortening velocity during the push-off of a vertical jump [63]. The slower fascicle shortening velocity in turn allows for greater force production according to the force–velocity relationship, while the faster rate of elastic energy recoil from the Achilles tendon enhances ankle power output, thus increasing jump height. Similarly, intermuscular coordination has also indirectly—by means of differences in sprint kinematics—been linked with sprint running speed [292]. Moreover, it has been suggested that the energy transporting role of biarticular muscles (from the hip to the knee) rather than force generation of individual muscles limits performance in cycling at high cadences (> 120 rotations per min) [293]. From an endurance perspective, suboptimal intermuscular coordination has also been linked to a reduced running economy [294] and lower lactate threshold (occurring at a lower wattage) during cycling [295]. A more optimal distribution of work over different muscles has also, for example, been observed in cyclists that have their lactate threshold at a higher percentage of their VO_2max_ [295]. Finally, altered intermuscular coordination has been (indirectly) associated with an increased injury risk in some sports [296–298].

Resistance training has the potential to alter intermuscular coordination and thereby indirectly impact performance and potentially affect injury risk [299, 300]. For example, three training sessions of maximal isometric arm flexor contractions reduced muscle inhibition of the biceps brachii short head from the synergistic brachioradialis muscle along with a 43% longer time to contraction failure at 20% elbow flexor maximal isometric contraction [300]. However, anecdotally some coaches and athletes believe that RT does not always improve, and in some cases may negatively affect, intermuscular coordination and thereby reduce athletic performance and increase injury risk. There is some experimental evidence to support the notion that some forms of RT may negatively affect intermuscular coordination. For example, isolated knee flexor training has been shown to produce greater knee flexor activation during an isolated knee extension task (i.e., increased antagonist co-activation; [301]), which theoretically could impair performance. Similar findings have been reported for isolated plantar dorsiflexion training [302]. Likewise, heavy resistance leg curl training has been shown to increase knee flexor co-contraction (and thus reduce net torque production) during a high-velocity isokinetic knee extension task, whereas high-velocity leg curl training reduced co-contractions [303]. Nevertheless, the majority of studies have demonstrated that antagonist muscle co-activation remains unaltered with RT [304–308], with some studies also reporting decreased coactivity [12, 309].

Further, some work also suggests that isolated training of muscles may impair coordination in multi-joint tasks. Computer simulations by Bobbert and Van Soest [287], for example, showed that increases in muscle strength without alterations in muscle onset and offset times (i.e., adjustments in intermuscular coordination) led to decreases in vertical jump performance in a musculoskeletal model. Similarly, Dalen and colleagues [291] experimentally showed that both single- and multi-joint training increased bilateral 1-RM squat strength, with a greater increase in 1-RM squat strength for the single-joint training group. However, despite a greater increase in 1-RM squat strength, the single-joint training group showed no change in vertical jump performance (and in fact a trend toward a decrease), whereas multi-joint training significantly increased vertical jump performance. An earlier study showed that training-induced alterations in intermuscular coordination could potentially explain these specific effects on jumping performance [310] as multi-joint training involving squatting with plantar flexion at the end of the squat led to more tightly coupled knee extensor and plantar flexor actions during vertical jumping while single-joint training (squat lift without plantar flexion plus separate calf raise exercise) led to more coupled hip and knee extensor muscle actions but more isolated plantar flexion, thus potentially reducing energy transport. Collectively, while high volumes of single-joint RT, as often employed in rehabilitation, or bodybuilding-type training may enhance both strength and muscle size (although the benefit over multi-joint exercises is mostly trivial to small [311]), they may not always improve complex movement performance. While any potential negative impact of single-joint training on performance is likely to be minimal within a comprehensive training program as compared to controlled studies, the above findings highlight that ‘isolated’ resistance training may negatively impact (or at least not improve) intermuscular coordination in some situations, and practitioners should carefully consider the specific effects of RT on intermuscular coordination and thereby on performance and, potentially, injury risk.

## Synthesis

In this review, we have highlighted that RT can trigger adaptations that may enhance both sprint and endurance (running) performances and reduce injury risk, while also highlighting several adaptations that may adversely impact performances and, potentially, increase injury risk. It is however important to note that RT results in adaptations other than those presented above. For example, neural adaptations such as reduced motor unit recruitment thresholds and reduced intracortical inhibition [2, 300, 312–315] may also have important beneficial effects on both endurance and sprint performances by increasing rate of force development and maximum force and power generation. Collectively, the beneficial adaptations to RT likely outweigh most, if not all, of the potential detrimental consequences discussed in the present review, in particular in relatively untrained individuals who are commonly the participants in research studies. For example, although there are trade-offs between strength and endurance adaptations (see also [316–318]), most studies [33, 75–77, 79, 81, 221, 319], but not all [81, 249, 320, 321], have reported improvements in movement economy or endurance capacity after periods of concurrent resistance and endurance training. Additionally, although there are trade-offs between maximizing strength and speed, a majority of studies have reported improvements in sprint performance after a period of RT [322]. Importantly, however, most of these studies have been performed over relatively short training periods (typically 12–18 weeks) and the long-term effects therefore require more investigation. Moreover, most studies included relatively untrained individuals, who are more likely to benefit from generic RT adaptations. However, some adaptive trade-offs may occur even in untrained individuals, such as increases in muscle fiber cross-sectional area that may attenuate increases in maximum oxygen uptake during concurrent training [89].

**Fig. 6:**
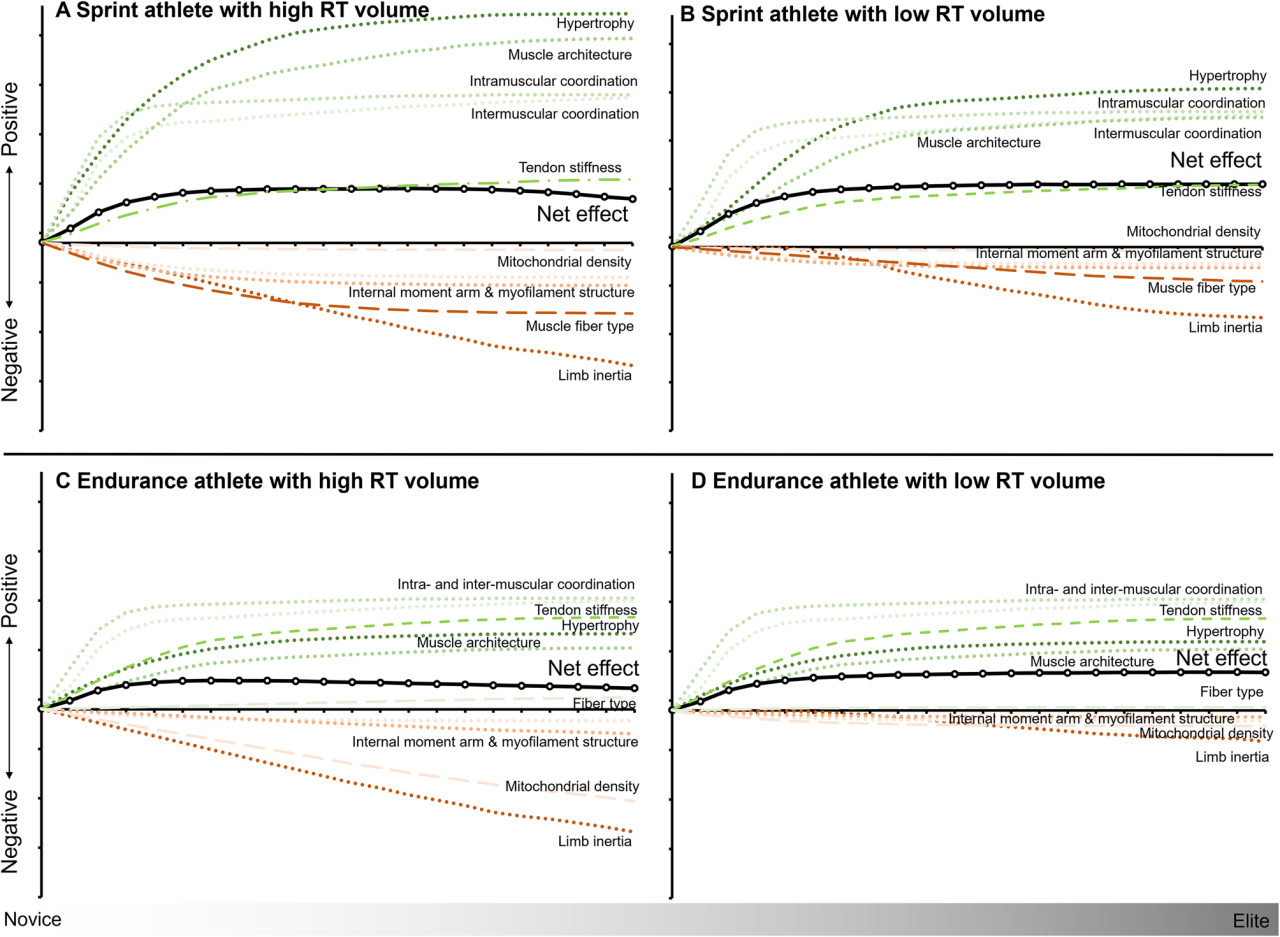
Balancing positive and negative adaptations to resistance training to optimize performance. Resistance training triggers numerous physiological adaptations, some which may benefit (green) or impair performance (red). The balance between beneficial and detrimental adaptations determines the net effect on sports performance (black solid line). An optimal resistance training program seeks to maximize beneficial adaptations while minimizing detrimental adaptations. For novice athletes, resistance training typically promotes numerous beneficial adaptations, resulting in rapid net improvements in performance. However, better trained athletes may have already gained most of the beneficial adaptations. While further performance improvements may be achieved in the short term through muscle hypertrophy, the increased limb inertia, potential alterations in fiber type, and increases in the internal moment arm that can accompany hypertrophic training practices may negate much of the beneficial effect of this increased hypertrophy, meaning highly trained athletes may require a more specific approach to training to optimize the benefit:cost ratio. Top: schematic for a sprint athlete who has not optimized his/her program (**A**; e.g., large volume of moderate load RT), or a sprint athlete who has optimized his/her program (**B**). The net beneficial effect eventually becomes detrimental in athlete A due to excessive muscle hypertrophy and resulting increases in body segment inertias. For athlete B, the net effect is larger due to smaller negative maladaptations and this athlete continues to show improvements over time. Bottom: a similar example for endurance running athletes, with endurance athlete **C** using a suboptimal program with a large number of sets and reps, that eventually may introduce excessive hypertrophy, resulting in a decreased net effect as compared to athlete **D** that has optimized his/her program. All changes are indicative only, and the relative importance should also be interpreted with caution as direct experimental evidence for the relative importance remains scarce or absent and likely may differ between sport disciplines and athletes

While the inclusion of a RT program is thus generally positive to both sprint and endurance performances, there are at least two reasons why it may be critical to consider potential maladaptations when designing a RT program. First, even if the net effect of adaptations is beneficial to performance or injury risk, minimization of any negative adaptations may improve the net beneficial effect (Fig. 6). For example, a sprint athlete may attempt to improve maximal motor unit discharge rates (and thus enhance RFD) by performing explosive-type, multi-joint resistance exercises [2, 323]. By minimizing the training volume and velocity loss during training sessions, shifts from type IIx to type IIa fibers may be minimized [4], potentially increasing the net beneficial effect of the training for fast force production. The lower training volume may also promote myofibrillar rather than sarcoplasmic hypertrophy (see Sect. 4), which will improve the strength (or power)-to-body mass ratio. This will in turn benefit performance since relative strength and power correlate better with sprint performance than absolute measures [19, 324–326], and some studies even report negative correlations between increases in body mass and sprinting performance following various training interventions [19, 20]. Finally, by using multi-joint exercises, athletes may also improve intermuscular coordination and reduce the potential for insufficient, excessive, or inappropriately timed co-activation of synergist and/or antagonist muscles [303] that could reduce net joint moments and thereby compromise performance.

A similar example can be provided for an endurance runner that aims to improve running economy by using three sets, each of five repetitions (~ 85% 1-RM load) of a heavy split-step squat combined with a heavy isometric calf hold. By using a low volume and heavy load, running economy can be improved more effectively than a higher volume low-load approach [281, 327, 328], which is typically used by endurance athletes [21, 23, 26]. Indeed, heavy RT leads to a balanced adaptation in muscle and tendon tissue [3, 189], which may not only better benefit running economy [182–184] but also reduce tendon injury risk [198, 329]. Moreover, the low training volume is less likely to induce substantial fatigue, which could otherwise impair the quality of a subsequent endurance training session.

A second reason for considering potential maladaptations is that well-trained athletes may have already gained some or most of the beneficial adaptations to training and may therefore benefit more from a specific training approach to optimize the ratio of beneficial to (potential) negative adaptations. For example, voluntary muscle activation is usually already high in young adults (> 85% or even > 95%, depending on the muscle and contraction type [1, 7, 313, 330]) and expectedly more so in physically active athletes, and is found to improve by ~ 5–10% with resistance training in only a few weeks [313, 330]. In support of this, recruitment and discharge rates of the biceps brachii could not explain the difference in maximum muscle force between long-term strength-trained individuals and untrained controls [331]. Additional RT therefore would have limited potential to further improve voluntary activation in individuals who already participate in some form of RT or sports in which voluntary activation improvements already occur. Further support for this is provided by a study showing stronger correlations between muscle volume and sprint/jump performances in better trained basketball players than lesser trained players [332]. The authors hypothesized that better trained players are optimized in terms of neural control, thus resulting in stronger correlations between muscle volume and performance as opposed to the lesser trained individuals in which variations in neural control may confound the relationship. Collectively, the evidence suggests that other adaptations such as muscle hypertrophy and adaptations in the extracellular matrix may be required to further increase maximal muscle strength, RFD, or power and thus to evoke improvements in sports performance. However, while a moderate amount of muscle hypertrophy will likely contribute positively to improved sprint and jump performances, there may be a point beyond which further increases have negative consequences, including promoting larger internal moment arms and larger limb and muscle inertias that may negate the beneficial effects of increasing muscle physiological cross-sectional areas. In support of this notion, increases in strength have been shown to contribute to improvements in sprint running speed up to some level only [19]. Similarly, plantar flexor muscle volume was greater in sprinters than body-size matched controls but did not differ between high and low performing sprinters [18]. Similar findings have been reported in other populations, with greater hypertrophy of selected muscles even being associated with reduced sprint running speed [37]. Therefore, it may be speculated that neural adaptations to RT contribute predominantly to improved performance in untrained individuals, whereas increases in muscle cross-sectional area (and type II fiber proportions, tendon stiffness) may contribute more dominantly in intermediately trained individuals, while subtle alterations in all of these (and other) mechanisms may contribute to performance in highly trained athletes (Fig. 6). In partial support of this scenario, vastus lateralis fascicle length and type IIa and IIx cross-sectional areas were found to be more strongly correlated with performances in some sports skills, including jump, sprint running, and shotput performances, in athletes with greater training experience, while the importance of muscle cross-sectional area and anthropometric characteristics decreased with smaller training experience [20]. In further support of considering trade-offs in adaptations, in particular for well-trained individuals, some studies have reported a trade-off between whole body force and velocity output that could partly reflect the discussed physiological trade-offs. For example, Colyer and colleagues [36] found that increases in the theoretical maximum propulsive ground reaction force following 6 months of training were associated with decreases in maximum velocity during sprinting, and vice versa, among elite skeleton athletes. Similarly, Bezodis and co-workers [35] found that larger RT volumes were associated with decreases in sprint performance/step frequency over the course of a periodized training program in elite sprinters.

## Practical Implications

The mechanisms discussed in this review have several potential implications for both sprint and endurance athletes, as discussed below. Based on the current scientific evidence, detrimental adaptations are most likely to be evoked when RT volumes are high in a combined endurance or sprint training program. Indeed, substantive muscle hypertrophy (and the associated increases in inertia, reduced mitochondrial density and alterations in microscopic structure such as filament spacing) may be more pronounced when exposed to large RT volumes, including when training is performed to failure [8, 112, 113]. Additionally, while myofibrillar and sarcoplasmic hypertrophy can both occur in response to low-volume RT, sarcoplasmic hypertrophy is more prominent after higher volume RT to failure, as typically done by body builders [45, 263]. Such training practices may reduce the ratio of force-to-cross-sectional area (or newtons-to-kilogram body mass ratio). Conversely, an increased myofibril packing density has been observed in power athletes who typically train with higher loads but with lower training volumes than body-builders [45]. Both sprint and endurance athletes may therefore benefit from a focus on low-volume, heavy RT without training to failure. Although there are no universal guidelines on the volume and intensity corresponding to low-volume, heavy RT, loads ≥ 85% 1-RM are typically regarded as heavy RT. This corresponds to a load that can typically be lifted six times. An athlete may however lift this load only four to five times per set to minimize fatigue by not training to failure, as this may disproportionally increase muscle mass relative to strength [113]. In endurance athletes, two to three sets per exercise with a total of two to three exercises per training session may then be used to maintain a relatively low RT volume [88]. For sprint athletes, a slightly higher volume may be used (e.g., three to four sets per exercise; three to four exercises per session), because relatively more muscle hypertrophy (causing a larger and hence stronger propulsive motor) may be beneficial for sprint and acceleration performance. However, this hypertrophy may be predominantly beneficial for proximal muscles as hypertrophy of distal muscle has a greater negative effect on limb inertia.

In further support of the use of heavy-load RT, the use of heavier training loads during RT does not necessarily lead to excessive muscle hypertrophy [8], while rather supporting neural [1, 271, 272, 333] and tendinous adaptations [3]. While a greater number of repetitions per exercise set (e.g., 12) is often performed in training based on the premise that high-threshold motor units will be better recruited (and thus trained) as the lower threshold motor units fatigue [334], neural drive and motor unit recruitment have been found to be reduced during moderate-load fatiguing repetitions compared to using high-load repetitions in some [335], although not all, studies [336]. Thus, it may not be possible to train the high threshold motor units equally effectively with low-load higher-repetition protocols. For endurance athletes, heavy-load RT therefore has been reported to be more beneficial for improving endurance performance [32–34] than low-load, higher-volume training RT protocols. Perhaps because of an historical fear of muscle mass gain, many endurance athletes who utilize RT, however, still use low-load training protocols with exercises performed to voluntary failure (e.g., back squat with 4 sets of 20 repetitions at 20-RM) [21, 23, 26]. For sprint athletes, heavy-load RT with low lifting volumes may also be more beneficial than performing low-load high-volume RT for additional reasons. Specifically, the conversion of type IIx fibers to type IIa fibers occurs when RT volume is high [5] or exercises are performed closer to failure [4]. In contrast, low-volume RT performed not to failure has been reported to (largely) conserve the proportion of type IIx fibers [4, 5, 233–235] and may therefore be better suited to improve RFD and enhance sprint performance. Nevertheless, the inclusion of periodized higher-volume, heavy-load RT may be required to increase muscle mass and strength, although at the expense of reduced type IIx fiber proportion. The increased RFD resulting from increases in maximum strength caused by preferential hypertrophy of type II myofibers [39] and enhanced neural drive to agonist muscles [271, 333] following such training may outweigh the detrimental effects of the decrease in type IIx proportions [337]. Moreover, tapering periods after such training blocks may trigger an overshoot in type IIx fiber proportion, which may benefit sprint performances [205], although the performance effects of this overshoot are yet to be documented. The overshooting in type IIx fiber proportions may explain why power outcomes show less decrement with training cessation than for example maximum strength [338]. Moreover, based on—albeit rare—observations that rate coding (maximal motor unit discharge rates) does not necessarily improve with heavy-load RT [2], sprint athletes may consider incorporating high-speed, maximal-effort (including ballistic) exercises to specifically train this quality. Finally, both sprint and endurance athletes are recommended to incorporate a significant proportion of multi-joint exercises within the training program to optimize intermuscular coordination.

Overall, both sprint and endurance athletes may benefit from a focus on low-volume, heavy-RT without training to failure, and/or using ballistic or plyometric exercise sets. Within this context, muscle hypertrophy may develop as a side-effect rather than a primary goal. Training methods such as velocity-based training [4, 339], cluster-set training [340] or using repetitions in reserve [113] may be useful in minimizing training exposure to failure and ensuring low-to-moderate training volumes to countermeasure potential detrimental adaptations. Note that such considerations may be more important for males than females due to the larger absolute muscle mass [341, 342] and thus potential negative side effects (e.g., inertia, internal moment arm) in males.

## Conclusions

This review highlights that RT adaptations are mostly, although not always, beneficial for improving both sprint and endurance (running) performances and reducing injury risk. For sprint athletes, beneficial adaptations will outweigh negative adaptations if RT is programmed with a relatively low volume that minimizes training to failure. Sprint athletes may also consider adding ballistic and plyometric exercises to improve rate of force development, and multi-joint exercises to optimize intermuscular coordination. Similarly, endurance athletes who perform high volumes of endurance training are unlikely to experience noticeable maladaptations when combining their training with a low-volume (high load) RT program. Instead, such a program is likely to induce adaptations that enhance performance. The beneficial effects of RT will be most pronounced among relatively untrained individuals, who are typically utilized in research studies, because athletes accustomed to RT may have already gained many of the beneficial adaptations and thus may be more at risk of inducing adaptations that can impair performance (e.g. excessive muscle or body mass). It follows from this that careful consideration and manipulation of RT training variables is important to optimize the sum of beneficial adaptations while minimizing detrimental adaptations in sprint and endurance athletes.
