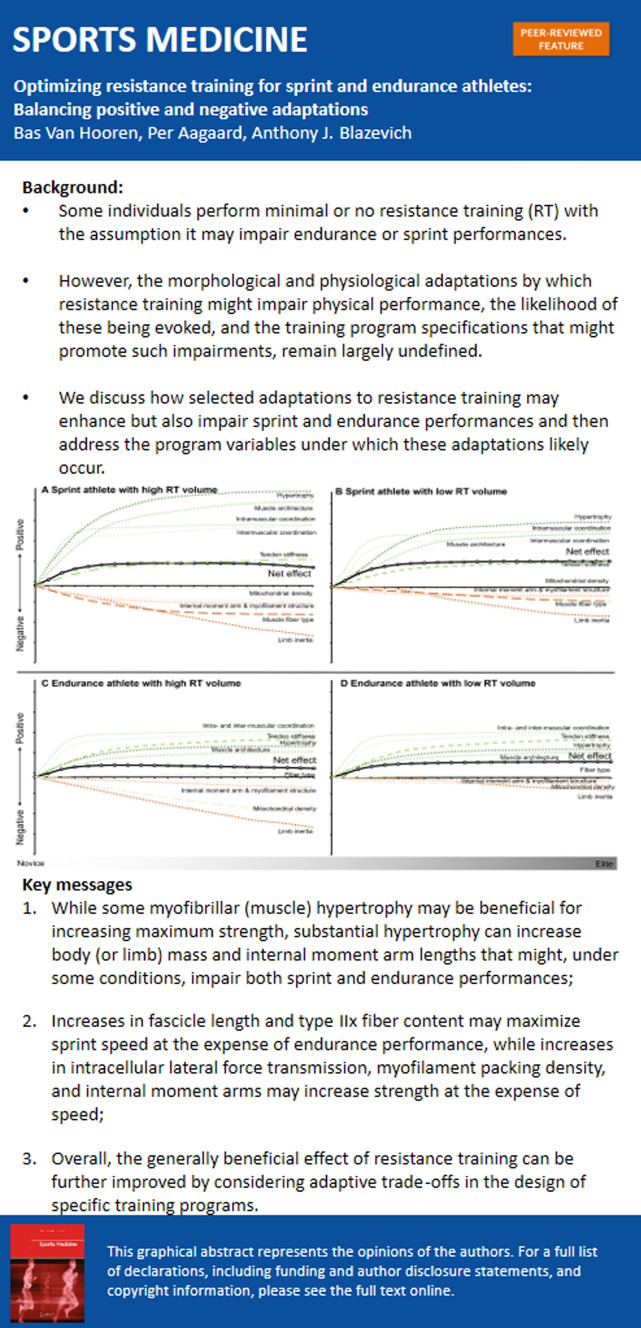# Correction to: Optimizing Resistance Training for Sprint and Endurance Athletes: Balancing Positive and Negative Adaptations

**DOI:** 10.1007/s40279-024-02162-6

**Published:** 2025-01-17

**Authors:** Bas Van Hooren, Per Aagaard, Anthony J. Blazevich

**Affiliations:** 1 Department of Nutrition and Movement Sciences, NUTRIM Institute of Nutrition and Translational Research in Metabolism, Maastricht University Medical Centre+, Universiteitssingel 50, Maastricht, NL 6229 ER The Netherlands; 2 Department of Sports Science and Clinical Biomechanics, University of Southern Denmark, Odense, Denmark; 3 Centre for Human Performance, School of Medical and Health Sciences, Edith Cowan University, Joondalup, Australia

**Correction to: Sports Medicine (2024) 54:3019–3050** 10.1007/s40279-024-02110-4

In this article the wrong figure appeared as Fig. 3 and Fig. 6; the Fig. 3 and Fig. 6 should have appeared as shown below.

**Figure 3:** 
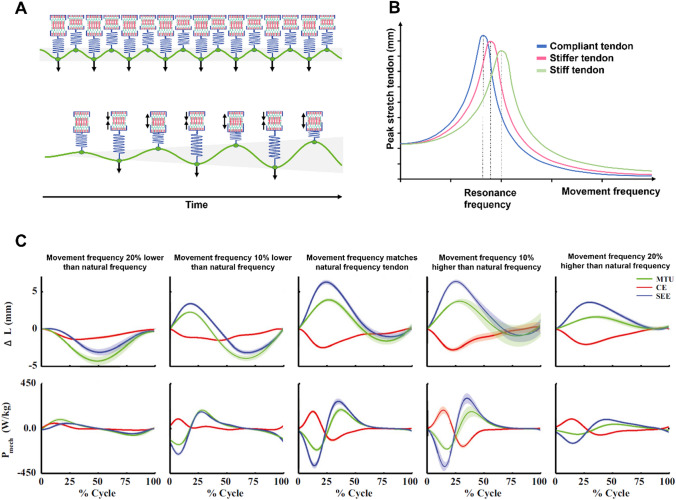


**Figure 6:** 
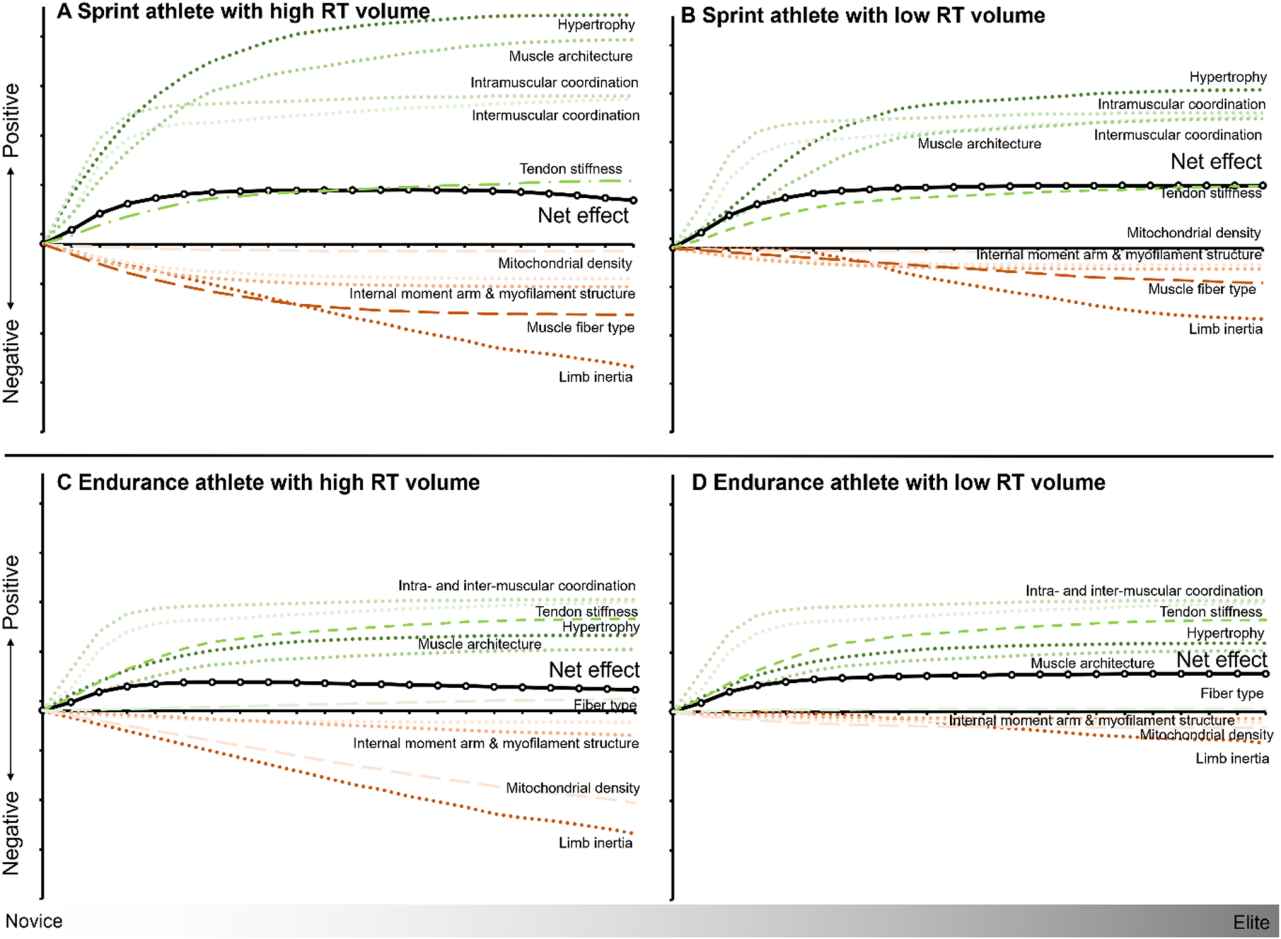


The graphical abstract should have appeared as below: